# Maintenance of stemness is associated with the interation of LRP6 and heparin-binding protein CCN2 autocrined by hepatocellular carcinoma

**DOI:** 10.1186/s13046-017-0576-3

**Published:** 2017-09-04

**Authors:** Qingan Jia, Yang Bu, Zhiming Wang, Bendong Chen, Qiangbo Zhang, Songning Yu, Qingguang Liu

**Affiliations:** 1 Department of Hepatobiliary Surgery, the First Affiliated Hospital of Xi’an Jiaotong University, 277 West Yanta Road, Xi’an, 710061 China; 2 Department of Hepatobiliary Surgery, General Hospital, Ningxia Medical University, Yinchuan, 750001 China; 3 Liver Cancer Institute, Zhongshan Hospital, Fudan University, Shanghai, 200032 China; 4 Department of General Surgery, Qilu Hospital, Shandong University, Jinan, 250012 China

**Keywords:** Hepatocellular carcinoma, CCN2, LRP6, Wnt, Combination therapy

## Abstract

**Background:**

The overall response rate of hepatocellular carcinoma (HCC) to chemotherapy is poor. In our previous study, oxaliplatin-resistant HCC is found to exhibit an enhanced stemness, and increased levels of CCN2 and LRP6, while the role of CCN2 and LRP6 in the prognosis of HCC patients, and the interaction regulation mechanism between CCN2 and LRP6 are still unclear.

**Methods:**

The expression levels of CCN2 and LRP6 were detected in large cohorts of HCCs, and functional analyses of CCN2 and LRP6 were performed both in vitro and in vivo. The roles of cell surface heparin sulfate proteoglycans (HSPGs) in the mutual regulatory between CCN2 and LRP6 were verified in HCC, and the interventions of low molecular weight heparin sodium (LMWH) were explored.

**Results:**

CCN2 and LRP6 were overexpressed in HCCs, and the CCN2 and LRP6 levels were positively associated with the malignant phenotypes and poor prognosis of HCCs. LRP6 could significantly upregulate the expression of CCN2. Meanwhile, CCN2 was able to enhance malignant phenotype of HCC cells in a dose-dependent manner through binding with LRP6; and knock-down of LRP6 expression, perturbation of HSPGs, co-incubation of CCN2 with LMWH could significantly block the adhesion of CCN2 to LRP6. LMWH enhanced the therapeutic effect of oxaliplatin on HCC with a high CCN2 expression.

**Conclusions:**

CCN2 plays a promoting role in HCC progression through activating LRP6 in a HSPGs-dependent manner. Heparin in combination with chemotherapy has a synergic effect and could be a treatment choice for HCCs with a high CCN2 expression.

**Electronic supplementary material:**

The online version of this article (doi:10.1186/s13046-017-0576-3) contains supplementary material, which is available to authorized users.

## Background

Hepatocellular carcinoma (HCC) is the fifth most frequently diagnosed cancer and the second cause of cancer death worldwide in men, with more than 80% of HCCs occurring in developing countries [1]. Even though much progression has been made in the clinical treatments of HCC, and many curative treatment strategies, such as surgical resection, radiofrequency, liver transplantation, and systemic chemotherapy have been proved to be useful in prolonging the survival of patients with HCC, the general prognosis for patients with HCC still remains extremely dismal, since fewer patients with advanced HCC can benefit from these curative treatments [2].

Chemoresistance as one of the critical malignant phenotypes of HCC, represents a major challenge in advanced HCC treatments, while, the exact mechanism remains not clear yet. The stem-like characteristics of cancer cells are thought to be one of the reasons. Like other kinds of solid tumors, HCC is hierarchically organized by a wide variety of cancer cells, including a subset of stem cells [3–6]. The cells with a more malignant potential contribute to the “stemness”, including maintenance of quiescence, sphere formation, high tumorigenicity, and resistance to hypoxia and chemoradiation [7]. Wnt signaling is one of the key pathways in regulating the cancer cell stemness [8]. Low-density lipoprotein receptor-related protein-6 (LRP6), which is a co-receptor in Wnt signaling, forms a signaling complex with Wnt ligand to activate downstream signaling. In HCC, aberrant expression and phosphorylation of cell surface LRP6 contributes to the activation of Wnt/β-catenin signaling pathway and play an important role in the hepatocarcinogenesis [9]. However, the role of LRP6 in the progression and prognosis of HCC patients is still unclear.

The CCN family is a small, six-member family of cysteine-rich regulatory proteins in humans, which share a multimodular structure with an N-terminal secretory signal domain followed by four conserved functional domains [10]. Therefore, CCN proteins not only behave like traditional growth factors or cytokines since it does not just appear to have a unique receptor to which it binds with high affinity to induce signal transduction. In the earlier years, several studies had described the role of CCN2 in proliferation, chemotaxis, adhesion, migration, and cell fate in different cell types and tissues [11–14]. By cDNA microarrays, we also found oxaliplatin-pretreated hepatocellular carcinoma exhibited the enhanced stemness and increased expression of CCN2 and LRP6 [15]. It was reported that LRP6 was one of the HSPGs-dependent adhesion receptors for CCN2, and co-incubation of CCN2 and heparin or perturbation of cell surface heparan sulfate proteoglycans (HSPGs) with heparinase completely blocked the adhesion of hepatic stellate cells to CCN2 [16]. Segarini also proved cells deficient for the receptor LRP6 were partly affected to bind with CCN2, with the notion that a principal function of CCN2 is to modulate receptor/ligand interactions [17]. In the field of cancer research, especially for HCC, the negative role of CCN2 and the interaction regulation mechanism between CCN2 and LRP6 are still unclear.

We hypothesize that LRP6 is positively regulated by CCN2, and is essential for the activation of the Wnt/β-catenin pathway directed by binding to the Wnt co-receptor LRP6 on HCC in a HSPGs-dependent manner, which is associated with enhanced stemness and poor prognosis in HCC. The study described herein demonstrated that malignant phenotype and poor prognosis were positively related to LRP6 and CCN2 in human HCC. And then, overexpression of LRP6 and CCN2 together was proved a major contributor to the enhanced stemness phenotype of HCC, and LRP6 could upregulated the expression of CCN2. Finally, this study showed that the mechanism of co-adhesion between LRP6 and CCN2 is dependent on cell surface HSPGs, highlighting the potential for heparin to be explored further as a therapeutic candidate for combination therapy in a subgroup of HCC patients with high expression of CCN2.

## Results

### CCN2 and LRP6 are up-regulated in human HCC and invasive HCC cell Lines

**Fig. 1:**
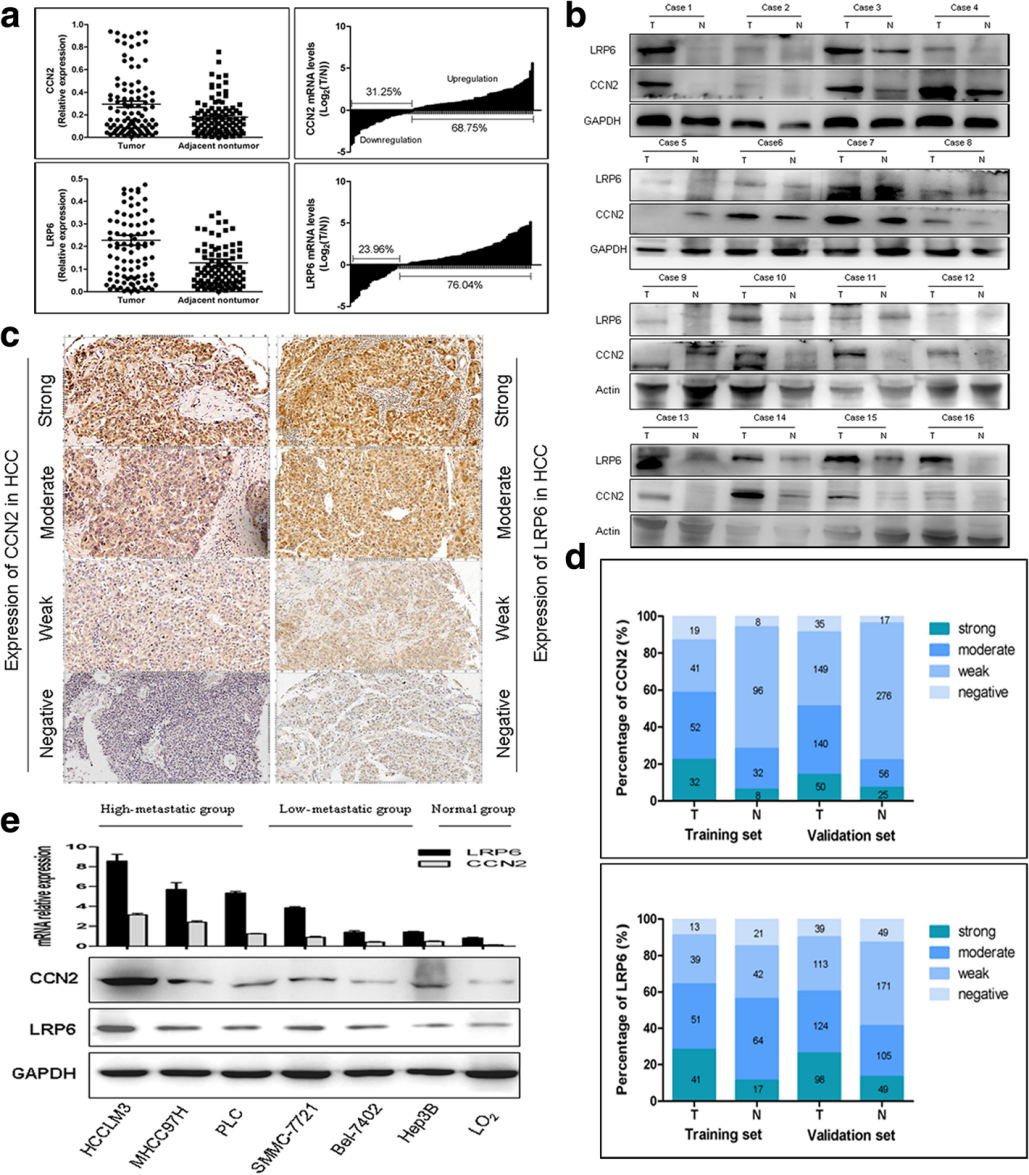
CCN2 and LRP6 are up-regulated in human HCC and invasive HCC cell Lines. **a** CCN2 and LRP6 mRNA expression in 96-paired HCC non tumor tissues. **b** Protein expression of CCN2 and LRP6 in tumor tissues (T) and adjacent nontumor tissues (N). **c** Representative immunostaining pictures of CCN2 and LRP6 in HCC patients. **d** Bar graph shows statistics for staining intensity in training (*N* = 144) and validation cohort (*N* = 374). **e** Relative CCN2 and LRP6 mRNA and protein levels in HCC cell lines and normal liver cell line L-02, and GAPDH was used as a loading control

To determine the role of CCN2 and LRP6 in HCCs, we first detected the mRNA expression levels of CCN2 and LRP6 in 96-paired HCC and adjacent non-tumor liver tissues. As compared with non-tumor liver tissues, up-regulation of CCN2 and LRP6 was observed in 68.75% (66/96) and 76.04% (73/96) HCC samples, respectively (Fig. 1a). Similar results in their proteins levels were found by Western blot in randomly selected 16 HCC samples (Fig. 1b) and immunohistochemical staining (IHC) in 144 HCC samples (Additional file 1: Figures S1 and S2). In IHC staining, positive staining for CCN2 was observed in cytoplasm, and LRP6 was observed in both cytoplasm and membrane (Fig. 1c). The alterations of CCN2 and LRP6 expression levels were further validated using IHC staining in tissue microarrays (TMA) containing tumor and non-tumor tissues from 374 HCC patients (validation cohorts). Although the CCN2 and LRP6 expression levels exhibited considerable heterogeneity in HCC tissues, the alteration tendency was consistent to the training set (Fig. 1d *;* Additional file 1: Figure S3A, B).

Previously, we found the up-regulated expression of CCN2 and LRP6 in oxaliplatin-resistant subcutaneous tumor mice screened by cDNA microarrays [15]. In the present study, increased expression levels of CCN2 and LRP6 were proved in oxaliplatin-resistant HCC cell lines and subcutaneous tumor tissues. [15] (Additional file 1: Figure S4). Increased CCN2 and LRP6 expression levels were also observed in seven HCC cell lines, especially in those with high malignant potential, whereas relatively lower expression levels were detected in liver cells (Fig. 1e).

### High expression levels of CCN2 and LRP6 are associated with malignant phenotype and poor prognosis of HCCs

To illustrate the clinical relevance of CCN2 and LRP6 expression in HCC, the 374 patients in the validation cohort were dichotomized according to high or low expression of CCN2 and LRP6. High expression of CCN2 was detected in 190 out of 374 HCCs (50.80%), which was significantly correlated with tumor number (*p* = 0.029), vascular invasion (*p* = 0.022), and tumor encapsulation (*p* = 0.034); however, no significant association was found between CCN2 expression level and the other clinical and pathological characteristics. On the other hand, high expression of LRP6 was found in 222 of 374 HCCs (59.35%), which was significantly correlated with vascular invasion (*p* = 0.016), and tumor encapsulation (*p* = 0.042); but no significant association was found between LRP6 expression and the other clinicopathological characteristics. (Additional file 2: Table S1).

**Fig. 2:**
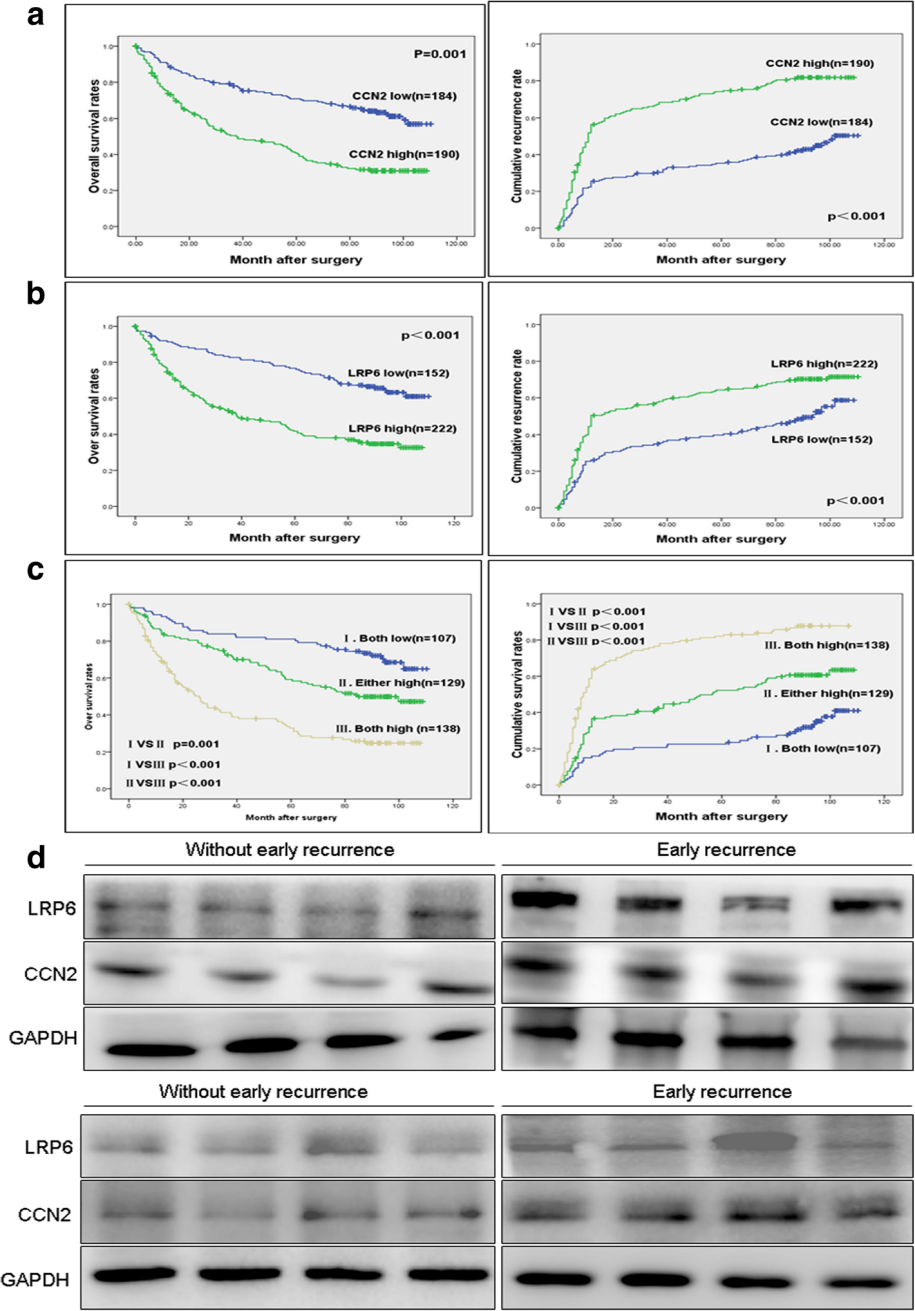
Up-regulation of CCN2 and LRP6 correlates with poor prognosis and in HCC patients. **﻿a**, **b**, **c**﻿ Kaplan-Meier’s curves for OS and TTR according to CCN2 and LRP6 expression in the validation cohort (*n* = 374). High expression levels of CCN2 and/or LRP6 are associated with poor prognosis of HCCs. **d** CCN2 and LRP6 expression in tumor with early recurrence (<24 months) and those without (*n* = 8)

In the CCN2^high^ group, the patients had significantly lower 5-year overall survival rates (OS) (35.26% vs. 63.15%, *p*<0.001) and higher 5-year cumulative recurrence rates (CCR) (64.21% vs. 32.60%, *p*<0.001) compared with those of the CCN2^low^ group (Fig. 2a). Similarly, the LRP6^high^-patients had a much lower 5-year OS (35.53% vs. 61.26%, *p*<0.001) and a higher CCR (60.81% vs. 29.61%, *p*<0.001) compared with those LRP6^low^-patients (Fig. 2b). Then, we classified the patients into three subgroups according to the different CCN2 and LRP6 levels: Group I had low expression levels of both CCN2 and LRP6 (*n* = 107); Group II had high level of either CCN2 or LRP6 (*n* = 129); and Group III had both CCN2 and LRP6 high expression (*n* = 138). The patients of Group I had the best prognosis, their 5-year OS rate was significantly higher than that of the patients in Groups II (70.09% vs. 51.94%, *p* = 0.001) and III (70.09% vs. 31.88%, *p*<0.000), and their CCR of Group I was significantly lower than the other two groups (75.70% vs. 56.59%, *p* < 0.001; 75.70% vs. 28.99%, *p* < 0.001, respectively; Fig. 2c).

Univariate analysis revealed that tumor size (*p* = 0.026; *p* = 0.019), tumor number (*p* = 0.010; *p* = 0.007), and vascular invasion (*p* = 0.003; *p* = 0.002), as well as the expression levels of CCN2 (*p* = 0.001; *p* = 0.004) and LRP6 (*p* < 0.001; *p* < 0.001) were significantly associated with the post-operative OS and CCR of HCC patients. AFP level (*p* = 0.003) was just associated with the CCR of HCC patients. However, no significantly prognostic significance was found in the other characteristics, including age, gender, HBsAg, HCV background, and liver cirrhosis (Additional file 2: Table S2). The multivariate Cox proportional hazards model revealed that overexpression of CCN2 (*p* = 0.002; *p* = 0.018) and LRP6 (*p* = 0.006; *p* = 0.014), vascular invasion (*p* = 0.019; *p* = 0.041), and tumor size (*p* = 0.013; *p* = 0.006) were independent prognostic indicators for OS and CCR of HCC patients; and AFP level (*p* = 0.039) was independent risk factor for the CCR of HCC patients. However, no significantly prognostic significance was found with the tumor number (Additional file 2: Table S3).

In 2003, Ye et al. [18] compared the gene expression profiles of 30 HCCs with or without metastasis. We reanalyzed the results and found LRP6 was significantly upregulated in HCC with metastasis (*p* = 0.0013) (Additional file 1: Figure S5). To further confirm the prognostic value of CCN2 and LRP6 level for HCC, we also analyzed them by immunoblotting in frozen tissue samples from HCC patients with and without early recurrence. Similarly, much higher levels of CCN2 and LRP6 expression were detected in HCC tissues with early recurrence compared with those non-recurrence HCCs (Fig. 2d).

### CCN2 and LRP6 enhance malignant phenotypes of HCC

**Fig. 3:**
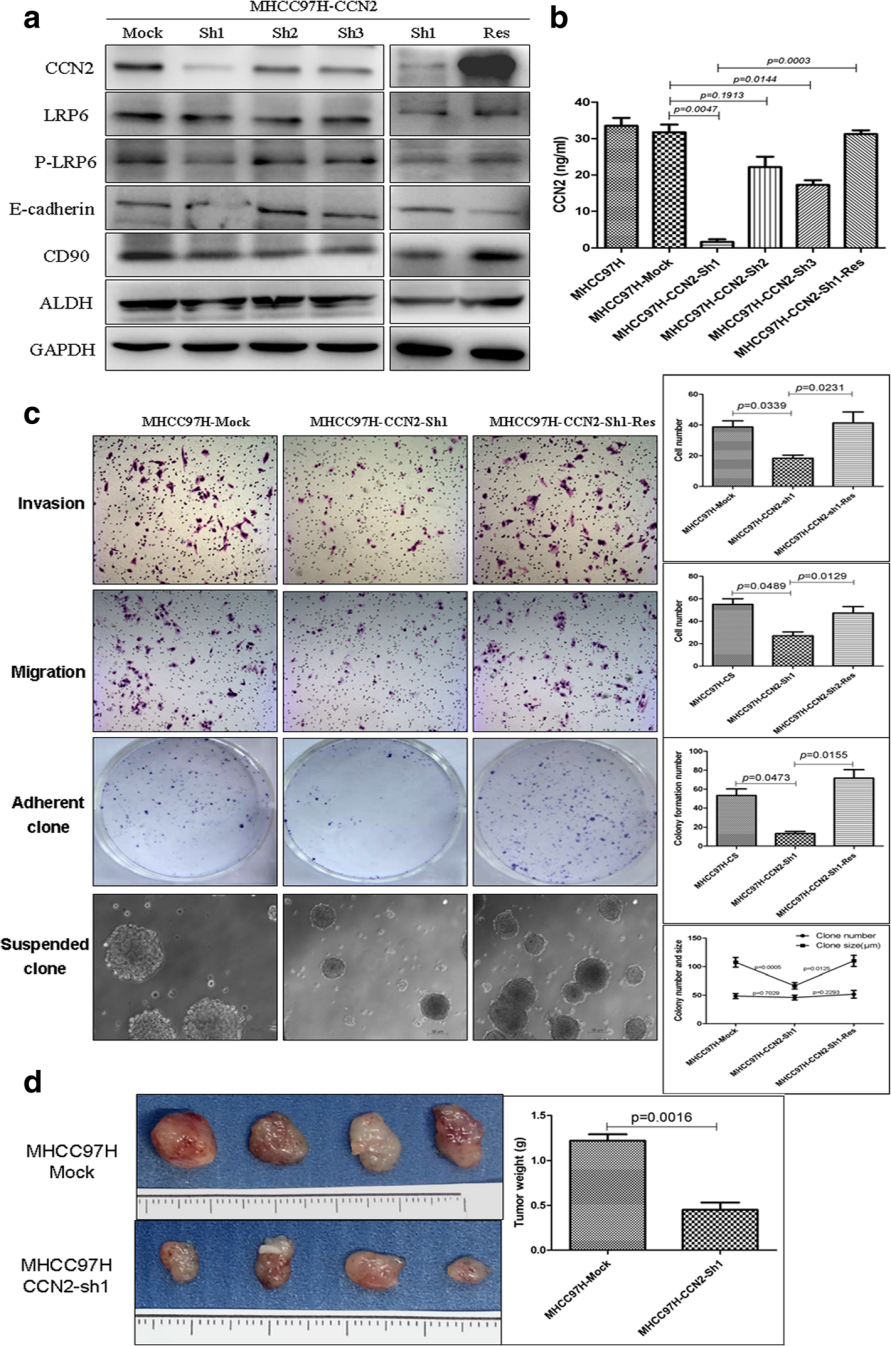
Expression of CCN2 in HCC is related to malignant phenotypes. **a** Endogenous CCN2 was silenced in MHCC97H cells using specific vshRNA. CCN2-Sh1 was validated as yielding the most efficient interference of CCN2 by western blot and ELISA. **b** Expression of LRP6, p-LRP6, E-cadherin, CD90, and ALDH, was determined following downregulation of CCN2 in MHCC97H cells by shRNA CCN2-Sh1 and gene rescue of CCN2. **c** Invasiveness, migration, adherent colony formation, and the anoikis ability were assessed among MHCC97H–Mock, MHCC97H-shRNA-CCN2 and CCN2 rescued cells. **d** Subcutaneous tumor growth capacity was determined for xenografts with MHCC97H–CCN2-sh1 cells or MHCC97H–Mock cells in nude mouse models

To evaluate the roles of CCN2 and LRP6 in HCC, first, we silenced the endogenous CCN2 expression in MHCC-97H cells. Among of the three CCN2-shRNA, CCN2-sh1 was found to exert the most efficient interference of CCN2 by immunoblotting and ELISA (1.65 ± 0.74 ng/ml vs. 31.76 ± 2.08 ng/ml *p* = 0.0047) compared with MHCC-97H-mock cells. Knock-down of CCN2 also induced the down-regulation of p-LRP6, CD90 and ALDH, but resulted in the up-regulation of E-cadherin. Restoration of CCN2 expression could rescue the altered expression of these proteins (Fig. 3a, b). In MHCC-97H-CCN2-sh cells, decreased CCN2 expression significantly impaired the invasiveness (18.33 ± 2.01 vs. 38.56 ± 4.02, *p* = 0.0339), migration (27.01 ± 3.45 vs. 55.00 ± 5.03, *p* = 0.0489) and proliferation (13.33 ± 2.91 vs. 55.73 ± 6.79, *p* = 0.0473) abilities compared with the controls. Furthermore, after rescue of CCN2 expression, the impaired invasiveness (41.33 ± 7.03 vs. 18.33 ± 2.01, *p* = 0.0231), migration (47.43 ± 5.61 vs. 27.01 ± 3.45, *p* = 0.0129) and cell proliferation (71.67 ± 8.97 vs. 13.33 ± 2.91, *p* = 0.0155; Fig. 3c) abilities of MHCC-97H-CCN2-sh cells were restored. Then, sphere formation ability was examined through suspension culture using MHCC-97H-CCN2-sh cells and CCN2 rescued cells. The decreased CCN2 expression significantly impaired the colony size (66.67 ± 5.73 μm vs. 107.51 ± 8.54 μm, *p* = 0.0005), while, rescue of CCN2 restored the impaired colony size (110.67 ± 9.66 μm vs. 66.67 ± 5.73 μm, *p* = 0.0125). There was no different in colony number (Fig. 3c). MHCC-97H-CCN2-sh cells also showed diminished subcutaneous tumor growth capacity compared with MHCC-97H-Mock cells in nude mouse models (0.47 ± 0.19 g vs.1.17 ± 0.22 g, *p* = 0.0016; Fig. 3d).

**Fig. 4:**
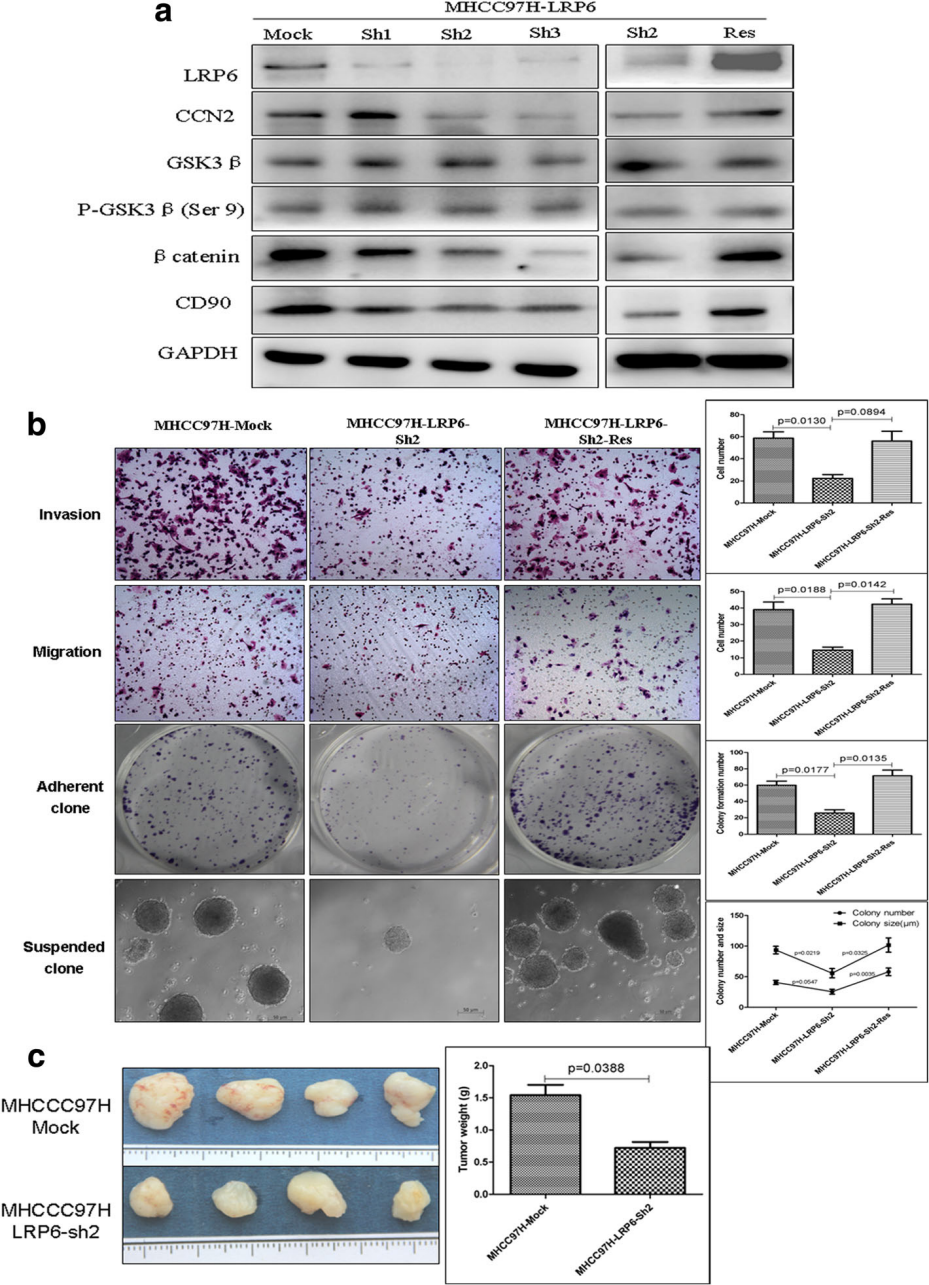
Expression of LRP6 in HCC is related to malignant phenotypes. **a** LRP6 was silenced in MHCC97H cells using specific vshRNA, and LRP6-Sh2 was validated as yielding the most efficient interference of LRP6 by western blot. Expression of CCN2 and β-catenin was determined following the downregulation of LRP6 in MHCC97H cells and following gene rescue of LRP6. **b** Invasiveness, migration, colony formation, and the anoikis ability were assessed among MHCC97H–Mock, MHCC97H-shRNA-LRP6, and cells and LRP6 rescued cells. **c** Subcutaneous tumor growth capacity of MHCC97H-shRNA-LRP6 cells and of MHCC97H–Mock cells in nude mouse model was assessed

Similarly, to examine the role of LRP6 in HCC, MHCC-97H cells were selected and successfully transfected with specific shRNA to silence LRP6 expression. Among the three LRP6-shRNA tested, LRP6-sh2 was able to induce the knock-down of LRP6 most efficiently. Knock-down of LRP6 led to a decrease in the expression of CCN2 and β-catenin in LRP6-Sh2-transfected MHCC-97H, and LRP6 restoration could reverse this alteration in protein levels (Fig. 4a). The invasiveness (22.33 ± 6.1 vs. 58.67 ± 10.021, *p* = 0.0130), migration (9.01 ± 7.68 vs. 14.67 ± 3.253, *p* = 0.0188), and proliferation (25.50 ± 8.27 vs. 59.75 ± 9.91, *p* = 0.0177) abilities of MHCC-97H-LRP6-sh cells were also significantly reduced compared to the controls. Furthermore, rescue of LRP6 could restore the impaired invasiveness (56.02 ± 15.51 vs. 22.33 ± 6.11, *p* = 0.0894), migration (42.33 ± 5.51 vs. 14.67 ± 3.25, *p* = 0.0142), and proliferation (71.25 ± 14.17 vs. 25.50 ± 8.27, *p* = 0.0135) abilities of MHCC-97H-LRP6-sh cells (Fig. 4b). The decreased LRP6 expression significantly impaired the colony size (55.83 ± 7.57 μm vs. 93.51 ± 6.19 μm, *p* = 0.0547) and colony number (25.5 ± 9.63 vs. 40.67 ± 8.28, *p* = 0.0219), while, rescue of LRP6 restored the impaired colony-forming ability with increased colony size (101.71 ± 11.67 vs. 55.83 ± 7.57, *p* = 0.0035) and colony number (58.00 ± 6.16 vs. 25.5 ± 9.63, *p* = 0.0325; Fig. 4c). The in vivo tumor growth capacity in subcutaneous implantation nude mouse models of MHCC-97H-LRP6-sh cells was also significantly diminished compared to the MHCC-97H-Mock cells (0.72 ± 0.18 g vs. 1.54 ± 0.32 g, *p* = 0.0388; Fig. 4d).

### CCN2 Enhances the Malignant Potential through upregulating Phosphorylation level of LRP6

**Fig. 5:**
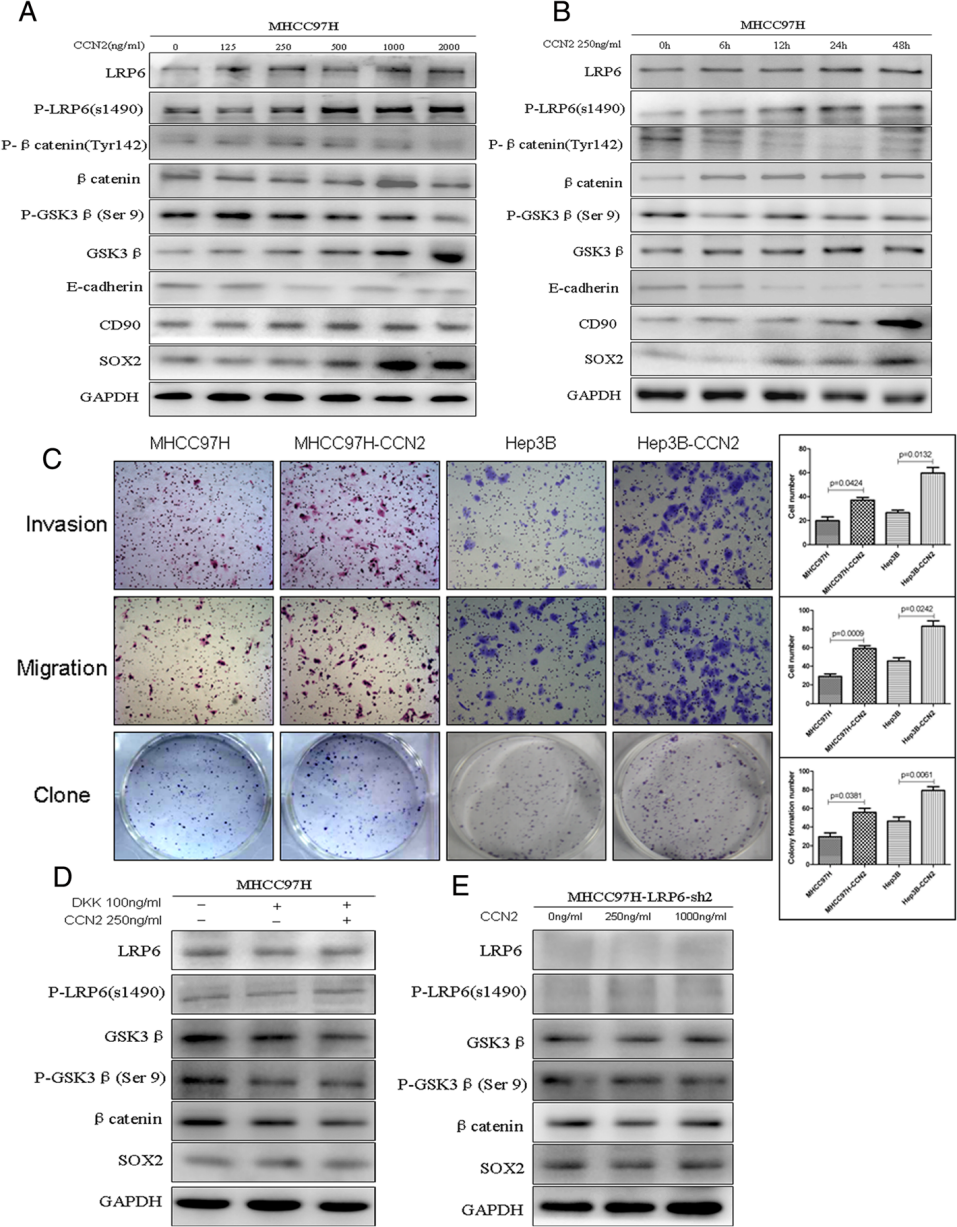
CCN2 activated Wnt signaling and enhanced malignant potential through the upregulation and phosphorylation of LRP6. Analysis of expression of LRP6 and p-LRP6, phosphorylation level of β-catenin, and expression of the stemness-related markers SOX2 and CD90 according to (**a**) increasing concentrations (0–2000 ng/ml) and (**b**) time (0–48 h) of CCN2. **c** Invasiveness, migration, and adherent colony formation were assessed in CCN2-treated HCC cell lines MHCC97H and Hep3B. **d** Expression of GSK3β, P-GSK3β (Ser9), β-catenin, and SOX2 was assessed in CCN2 (250 ng/ml)-treated MHCC97H cells following treatment with the specific LRP6 inhibitor DKK1 (100 ng/ml) for 24 h. **e** Wnt signaling associated proteins GSK3β, P-GSK3β (Ser9), β-catenin, and SOX2 were assessed in the LRP6-knockdown cell line MHCC97H–LRP6-Sh2, which was treated with CCN2 in increasing concentrations (0, 250, 1000 ng/ml)

To investigate the effect of CCN2 on Wnt signaling, we treated HCC cells with recombinant CCN2, and found that CCN2 was able to activate Wnt signaling with concomitant upregulation of LRP6, phosphorylated LRP6, and β-catenin, but decrease the phosphorylation level of β-catenin. It can also induce the upregulation of the stemness-related markers SOX2 and CD90, and downregulation of E-cadherin. These effects were in both a dose-dependent manner (0–2000 ng/ml; Fig. 5a) and a time-dependent manner (0–48 h; Fig. 5b). CCN2 treatment could also significantly enhance the invasiveness (37.00 ± 4.55 vs. 19.75 ± 6.40, *p* = 0.0424), migration (59.04 ± 5.94 vs.29.00 ± 5.48, *p* = 0.0009), and proliferation (55.75 ± 8.737 vs.29.50 ± 8.2, *p* = 0.0381) abilities of MHCC-97 cell lines (Fig. 5c); Similarly, significant increases in invasiveness (59.75 ± 9.43 vs. 26.50 ± 4.20, *p* = 0.0132), migration (83.01 ± 11.63 vs. 45.50 ± 7.19, *p* = 0.0242), and proliferation (79.24 ± 8.61 vs. 46.25 ± 9.11, *p* = 0.0061) abilities were observed when Hep3B cells was treated with CCN2 (Fig. 5c).

In addition, the specific LRP6 inhibitor DKK1 was used to examine the role of LRP6 in Wnt activation induced by CCN2. DKK1 treatment significantly inhibited the Wnt signal, and CCN2 couldn’t significant reverse the downward trend without any change of p-LRP6, GSK3β, P-GSK3β (Ser9), β-catenin, or SOX2 levels (Fig. 5d). To further clarify the role of LRP6 in Wnt signal activation, the LRP6 knockdown cell MHCC-97H-LRP6-Sh was treated with CCN2 in step-increasing concentrations, but no any significant change in Wnt signaling was observed (Fig. 5e).

### CCN2 Binds with LRP6 in a HSPGs-dependent Manner, and co-incubation of CCN2 with LMWH blocks the binding

**Fig. 6:**
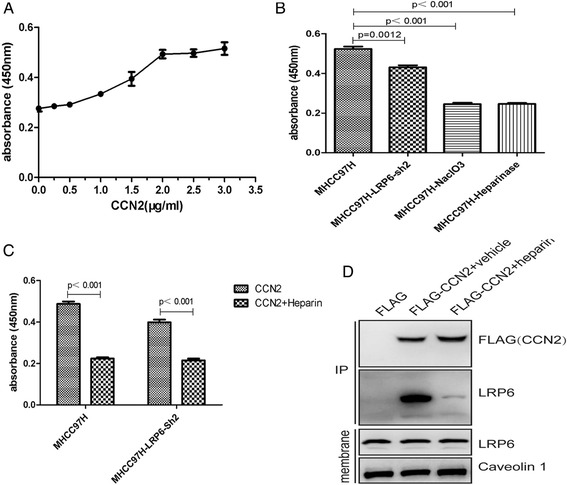
CCN2 binding with LRP6 occurs in a HSPGs-dependent Manner. **a** Dose-dependent promotion of adhesion of HCC cells was determined by incubation of MHCC97H cells in 96-well plates that had been pre-coated with 0–3 μg/ml recombinant human CCN2. **b** MHCC97H–LRP6-Sh2 exhibited decreased cell adhesion to CCN2 compared to untreated MHCC97H cells, and destruction of HSPGs with heparinase or inhibition of HSPG sulfation with NaClO_3_ exhibited almost complete inhibition effect for MHCC97H adhesion to CCN2. **c** Adhesion of HCC cells was determined following co-incubation of CCN2 with heparin. **d** LMWH inhibited the interaction between CCN2 and LRP6

In the present study, dose-dependent promotion of adhesion was observed in MHCC-97H cells when they were incubated in 96-well plates that had been pre-coated with 0–3 μg/ml recombinant human CCN2 (Fig. 6a). To determine the potential role of LRP6 on cell surface in CCN2-mediated adhesion, we found that MHCC-97H-LRP6-sh cells exhibited a significant decrease in cell adhesion of compared to untreated MHCC-97H cells (0.43 ± 0.02 vs. 0.52 ± 0.03, *p* = 0.0012). Adhesion of MHCC-97H cells to CCN2 was significantly blocked after the destruction of HSPGs with heparinase (0.25 ± 0.01 vs. 0.52 ± 0.03, *p* < 0.001) or inhibition of HSPG sulfation with NaClO_3_ (0.25 ± 0.02 vs. 0.52 ± 0.03, *p* < 0.001) (Fig. 6b). In addition, co-incubation of CCN2 with low molecular weight heparin (LMWH) significantly blocked the adhesion of MHCC-97H cells (0.22 ± 0.01 vs. 0.49 ± 0.03, *p* < 0.001) and MHCC-97H-LRP6-sh cells (0.22 ± 0.02 vs. 0.40 ± 0.03, *p* < 0.001) to CCN2 (Fig. 6c).

In addition, to gain more insights on the CCN2-LRP6 interaction, HCC-97H cells were transfected with Flag-CCN2 or empty vector. Co-IP assays showed CCN2 formed a complex with Wnt signal co-receptor LRP6, and this complex could be significantly blocked by LMWH (Fig. 6d).

### LMWH Enhances the therapeutic effect of oxaliplatin on HCC with high expression of CCN2

**Fig. 7:**
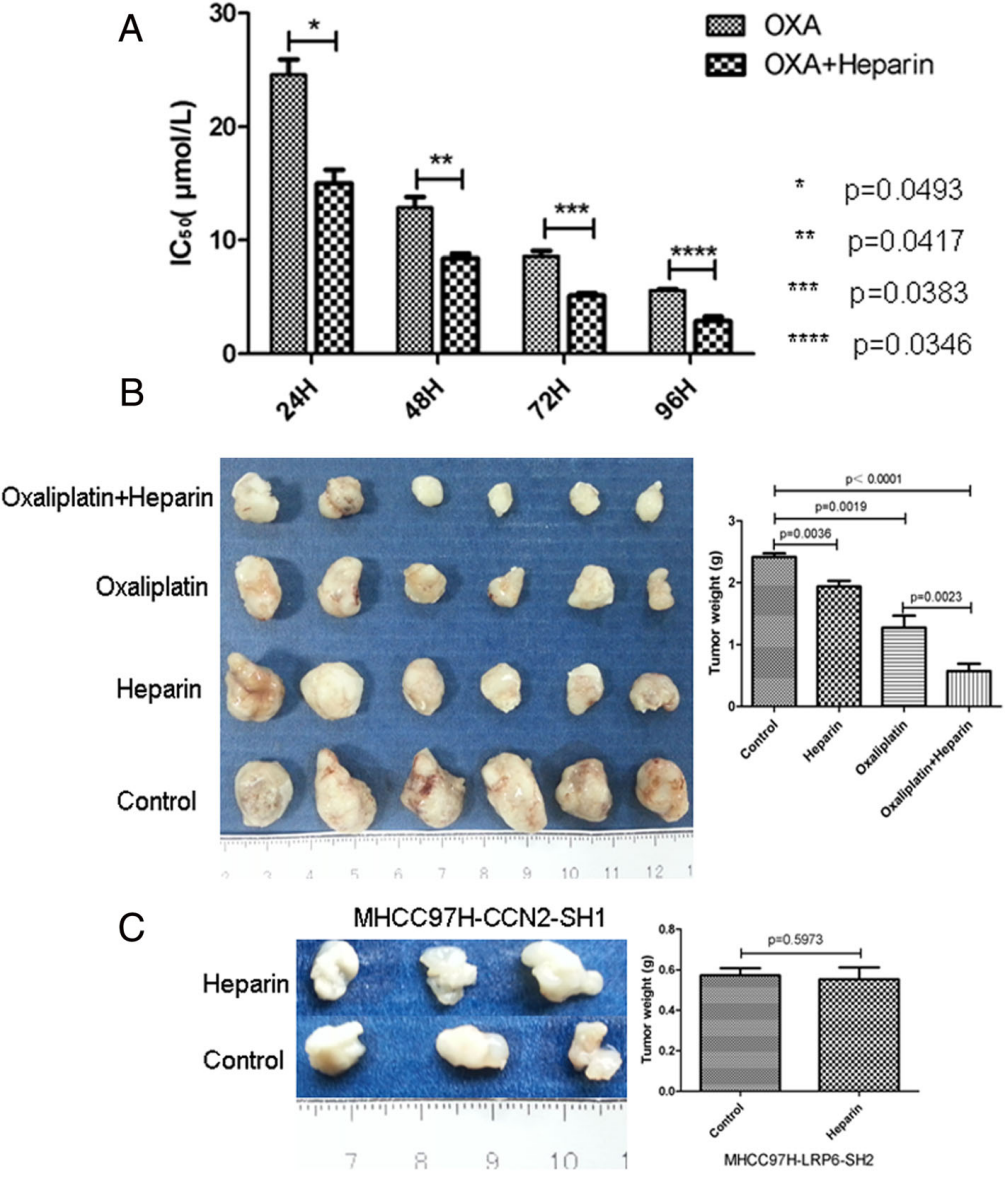
Low molecular weight heparin sodium (LMWHs) inhibited tumor growth, especially in cell lines that exhibit high expression of CCN2, and combination treatment with LMWHs and oxaliplatin resulted in an enhanced chemotherapeutic effect. **a** Proliferation was assessed for MHCC97H cells that were treated with LMWHs (2 U/mL) for 24, 48, 72, or 96 h with or without oxaliplatin. **b, c** Tumor proliferation was examined in mouse xenografts established with MHCC97H cells with high expression of CCN2 or with low expression of CCN2, and then treated with LMWHs or with LMWHs and oxaliplatin

We used LMWH (2 U/mL) to treat MHCC-97H cells for 24, 48, 72, or 96 h, and evaluated its synergetic effect with chemotherapy. We found that LMWH alone didn’t demonstrate significant inhibitory effect on the in vitro proliferation of MHCC-97H cells (Additional file 1: Figure S6), but significantly increased the sensitivity MHCC-97H cells to oxaliplatin. The IC50 of oxaliplatin in combination with LMWH was significantly decreased compared with that of oxaliplatin alone (treatment for 24 h, 15.01 ± 2.06 vs. 24.57 ± 2.32, *p* = 0.0493; for 48 h, 7.82 ± 0.72 vs. 13.42 ± 1.37, *p* = 0.0417; for 72 h, 5.13 ± 0.41 vs. 8.59 ± 0.81, *p* = 0.0383; and for 96 h, 2.90 ± 0.67 vs. 5.58 ± 0.21, *p* = 0.0346) (Fig. 7a *;* Additional file 1: Figure S7).

To further evaluate the synergetic effect of oxaliplatin with LMWH on in vivo tumor growth, we established subcutaneous xenograft models using MHCC-97H cells. The mice models were divided into four groups: the controls, LMWH alone, oxaliplatin alone, and LMWH +oxaliplatin group. Interestingly, LMWH alone could inhibit the in vivo tumor growth of MHCC97H cells (with high CCN2 expression) compared to the controls (2.42 ± 0.14 vs. 1.94 ± 0.23, *p* = 0.0036), but exhibited no significant inhibitory effect on tumor growth of MHCC-97H-CCN2-Sh cells (0.58 ± 0.06 vs. 0.55 ± 0.12, *p* = 0.5973) (Fig. 7c). Moreover, the combination of LMWH (250 U/kg) significantly enhanced the inhibitory effect of oxaliplatin, as evidenced by the much lower tumor weights in the heparin + oxaliplatin group compared with the oxaliplatin alone group (0.57 ± 0.28 vs. 1.28 ± 0.47, *p* = 0.0023), although oxaliplatin alone also induced a significant antitumor effect (1.28 ± 0.47 vs. 2.42 ± 0.14, *p* = 0.0019; Fig. 7b).

## Discussion

For HCC patients diagnosed at early stages, potentially curative treatments are available, such as radiofrequency ablation, resection, and liver transplantation. While, more than 70% of patients are at an advanced stage when HCC diagnosed, and are not eligible for curative therapy. Therefore, transcatheter hepatic arterial chemoembolization (TACE) and systemic chemotherapy are the most common methods of treatment. Unfortunately, the overall response rate of HCC to such treatments is poor, due in part to the relatively high stemness of these cancer cells [15]. And the reducing of stemness may have the potential to favourably make up for the deficiencies of treatment options and thus benefit the patients.

In cancer, Wnt signaling is one of the key signaling pathways related to stemness, which is frequently activated, and plays an important role in hepatocarcinogenesis and malignant progression. Activation of Wnt/β-catenin signaling results in β-catenin translocation into the nucleus, where this factor activates target genes that regulate stemness. LRP6, which is a co-receptor for Wnt ligand and this complex, activates Wnt downstream signaling [19, 20]. In 2003, our group collaborated with the United States’ National Cancer Institute to compare the gene expression profiles of HCC with or without metastasis, and found LRP6 was significantly upregulated compared with the liver tissue of nonmetastatic HCC [18]. Overexpression of LRP6 in HCCs was also proved in the present study, and which was positively associated with malignant phenotypes and poor prognosis of HCC patients. Then, we found the increased expression of LRP6 in oxaliplatin-resistant hepatocellular carcinoma with enhanced stemness. In HCC cell lines, we also proved enhanced stem-like characteristics of cancer cells were related to high expression of LRP6. Meanwhile, we also found there was a positive relationship between the phosphorylation of LRP6 and CCN2. Then, the negative role of CCN2 and the mutual regulatory mechanism between CCN2 and LRP6 were explored in HCC.

The CCN family, first described by P. Bork in 1993, is a small, six-member family of cysteine-rich regulatory proteins found in humans, which share a multi-modular structure with an N-terminal secretory signal domain followed by four conserved domains, including an IGF binding domain (IGFBP), a von Willebrand type C domain (VWC), a thrombospondin-1 domain (TSP1), and a cystine knot domain (CT) [21]. Cause of their four conserved domains, they can modulate the activities of many peptide growth factors [22, 23]. CCN2 as one of the CCN family proteins has been implicated in various biological processes including cell migration and tumor progression [24]. In diabetic nephropathy, the activation of Wnt signaling in mesangial cells by CCN2 was along with stimulated phosphorylation of LRP6, nuclear localization of β-catenin, and expression of Wnt targets [25]. Previously, we had proved oxaliplatin-resistant HCC exhibited increasing pulmonary metastatic potential with 267 significantly up-regulated genes by DNA microarray analysis, including CCN2 [15]. Thus, in HCC, we speculate CCN2 could regulate Wnt signaling pathway probably because of its ability to bind to the Wnt co-receptor LRP6. In the present study, we confirmed the increased expression of CCN2 in oxaliplatin-resistant hepatocellular carcinoma with enhanced stemness. And the downregulation and rescue of CCN2 altered the phosphorylation level of LRP6, as well as the expression of the associated downstream Wnt signaling factors. The enhanced stemness and the related biomarkers were also studied after the upregulation or overexpression of CCN2, and our findings support the notion that CCN2 is responsible for LRP6 receptor interactions. And inhibition of CCN2 could downregulate Wnt signaling and inhibite the stemness of HCC. According to the structure characteristic, LRP6 is one of the HSPGs-dependent adhesion receptor for CCN2 [10, 26]. Thus, it will be very exciting to understand the associated mutual regulation of CCN2 and LRP6 in HCC, and such information may underscore a novel implication of heparin in anti-HCC therapy.

The diverse but specific interactions of CCN2 with cell surface receptors permits their participation in a broad spectrum of cellular processes, whereas cell surface HSPGs serve as binding sites for all CCN family proteins, including CCN2. Gao et al. [16] demonstrated that co-incubation of CCN2 with heparin or perturbation of cell surface HSPGs with heparinase completely blocks the adhesion between CCN2 and hepatic stellate cells. In the present study, we also proved dose-dependent adhesion of recombinant human CCN2 to an HCC cell line MHCC97H. The role of LRP6 on the HCC cell surface in CCN2-mediated adhesion was also demonstrated. Adhesion of HCC cells to CCN2 was nearly completely blocked after the destruction of HSPGs with heparinase or inhibition of HSPGs sulfation with NaClO3. Co-incubation of CCN2 with heparin also completely blocked the adhesion between LRP6 and CCN2. Together, these results indicated that CCN2 binding with LRP6 is a HSPGs-dependent process in HCC, and these findings are critical for us to develop treatment regimens to downregulation of Wnt signaling and inhibition of stemness of HCC, for those with high expression of CCN2.

**Fig. 8:**
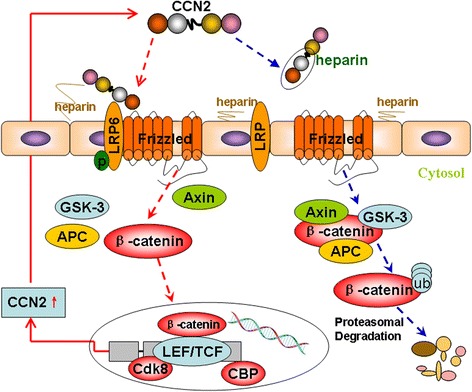
Cell surface HSPGs consists of a protein core linking several linear heparan sulfate (HS) chains. HS chains ensure CCN2 bind to the cell surface, and decisively regulate their accessibility, LRP6 phosphorylation, and Wnt activation. Co-incubation of LMWH with CCN2 could significantly block the adhesion of CCN2 to cell surface HSPG, and inhibit the function of CCN2. Oxaliplatin could upregulate CCN2 expression and activate Wnt signaling, while, co-incubation of CCN2 with heparin also completely blocked the adhesion between LRP6 and CCN2, and activation of Wnt signaling pathway

In clinical trials with cancer patients, low molecular weight heparin sodium (LMWH) appears to prolong survival of patients with advanced malignancy [27–29]. Altinbas et al. [30] found small cell lung cancer (SCLC) was a chemotherapy responsive tumor and associated with alterations in the coagulation system, and addition of LMWH to combination chemotherapy resulted in increase in survival. Jong et al. [31] found that LMWH use was an independent predictor of improved survival in men with metastatic castration resistant prostate cancer receiving docetaxel. Our research group also proved LMWH could inhibit tumor growth and metastasis by inhibiting tumor angiogenesis in nude mice HCC models [32]. Evidence suggests that heparin species inhibit mitogenic signaling mainly through inhibition of growth factors and their receptors [33], and/or by inhibition of the enzyme heparanase [34]. Another possibility is that heparin inhibits metastasis by blocking platelet-tumor cell interactions, thereby inhibiting aggregates of tumor cells lodging in the microvasculature. In the present study, we demonstrated LMWH exhibited no significant proliferation inhibition but showed increased sensitivity to oxaliplatin when combined with LMWH. Interestingly, LMWH partially inhibited the tumors that had been established with MHCC97H cells with the high expression of CCN2, but did not significantly inhibit proliferation of tumors established with MHCC97H–CCN2-Sh cells. Through our research, this anti-tumor effect of LMWH in this study may be contributed to the interfering core regulatory functions of CCN2 proteins that function to orchestrate the Wnt co-receptor LRP6 (Fig. 8).

## Conclusions

In conclusion, we have demonstrated that CCN2 plays an adverse role that is directed by the phosphorylation of the Wnt co-receptor LRP6 in HCC in a HSPGs-dependent manner. Combination treatment with oxaliplatin and LMWH resulted in an enhanced chemotherapeutic effect on HCC tumors with high expression of CCN2. While, the utility of heparin and LMWH as anticancer drugs is limited due to their anticoagulant activity, non-anticoagulant heparins are preferable for potential clinical use because they could be administered at high doses, thereby fully exploiting the antimetastatic component of heparin, and because they could be applied to cancer patients with bleeding complication, such as HCC.

## Methods

### Cell lines and animals

The human HCC cell lines with high metastatic potential used in this study were HCCLM3 and MHCC97H cells (established at Fudan University) [35], and which were supplied and authenticated in the year 2010 during study initiation by Biosyn, Inc. using DNA profiling of short tandem repeat markers. The human HCC cell lines with low metastatic potential were SMMC-7721 cells (established at Second Military Medical University), and PLC, Bel7402, and Hep3B cells (obtained from American Type Culture Collection), and the human liver cell line LO2 (obtained from Chinese Academy of Science) were all conserved and supplied by our Liver Cancer Institute in the year 2010 during study initiation, and no authentication was done. All cells were maintained in DMEM (GICBO, Grand Island, NY) supplemented with 10% fetal bovine serum (GICBO) at 37 °C in a humidified incubator with 5% CO_2_. It was routinely screened for presence of mycoplasma (Mycoplasma Detection Kit, Roche Diagnostics) during the study period.

Male BALB/c nu/nu mice (aged 4–6 weeks and weighing approximately 20 g) were obtained from the Chinese Academy of Science (SLRC, Shanghai, China) and maintained under standard pathogen-free conditions. The experimental protocol was approved by the Shanghai Medical Experimental Animal Care Commission.

### Patients and follow-up

A total of 630 tissue specimens were obtained for this study. In training set, 104 paired HCC samples were used for immunohistochemistry, 96 paired HCC samples were used for real-time PCR, and 16 paired HCC samples were used for Western blot. In validation set, 374 patients who underwent curative resection between January 2004 and December 2006 at the Liver Cancer Institute, Zhongshan Hospital, Fudan University provided the samples for immunohistochemistry. 374 Patients were followed after surgical treatment until December 2013, and the median follow-up was 63 months (range, 0–110 months). The follow-up procedures were described in detail in a previous report [36]. Curative resection was defined as complete resection of tumor nodules, leaving tumor margins free of cancer upon histologic examination. The histopathologic diagnosis was based on World Health Organization criteria. The detailed clinicopathologic characteristics of all HCC study patients in the study are listed in Additional file 2: Tables S1–S3.

Ethical approval was obtained from the Zhongshan Hospital Research Ethics Committee, and informed consent was obtained from each patient.

### Vector construction, transfection and lentivirus transduction

The human full-length cDNA of CCN2 (NM_001901.2) and LRP6 (NM_002336) were obtained from Genesent (shanghai China) and then cloned into the pCDH lentiviral expression vector (System Biosciences). Using the In-Fusion HD Cloning Kit (Takara), the amplified fragment was inserted into the plasmid pCDH (between XbaI and EcoRI sites). Flag-tagged CCN2 in pCDH vector was from Genesent (shanghai China). The primers were listed in Additional file 2: Table S6. Lentiviral shRNA expression plasmids PLKO.1, three different shRNAs against CCN2 and LRP6 mRNA are listed in Additional file 2: Table S7.

### Cell binding assays

CCN2 was diluted in PBS and used to coat 96-well plates (50 μl/well) for 20 h at 4 °C. Wells were then blocked with 1% BSA for 1 h at 37 °C. A volume of 50 μl of cell suspension containing 1.5 × 10^4^ MHCC97H cells was added to each well for 20 min at 37 °C, and then the wells were washed three times with 200 μl PBS. Alternatively, 2 μg/ml heparin was mixed with MHCC97H cells just prior to plating. Adherent cells were counted using the Cell Counting Kit 8 (Dojindo,Kumamoto, Japan).

### Immunoprecipitation assay

For purifying Flag-tagged CCN2 fusion proteins, HEK-293 T cells with Flag-tagged CCN2 stable overexpression were harvested in RIPA lysis buffer supplemented with complete protease inhibitor and phosphatase inhibitor (Roche Applied Science). The lysate was cleared by centrifugation at 12000 g before being loaded to M2 anti-Flag mAb agarose beads (Sigma, St. Louis, MO, USA) pre-equilibrated in RIPA buffer overnight at 4 °C. The beads were washed with RIPA buffer five times and 30ul RIPA buffer was added to cover the beads. The RIPA buffer containing beads and Flag-tagged CCN2 fusion proteins were stored at 4 °C for the next experiment. For the extraction of membrane proteins, MHCC97H cells were grown to 75% confluence. The membrane proteins were extracted using a ProteoExtract Native Membrane Protein Extraction Kit (M-PEK Kit; Calbiochem, La Jolla, CA, USA) according to manufacturer’s instruction. For the binding assay, Flag-tagged CCN2 bind to the beads was incubated with membrane proteins with or without LMWH (2 μg/ml, Santa Cruz Biotechnology) overnight at 4 °C in RIPA buffer. Then, the beads were washed with RIPA buffer five times and bound proteins eluted using Flag peptide (Sigma). The washed protein was boiled in loading buffer, resolved on SDS-PAGE. Subsequent immunoblots were probed with the appropriate antibody and detected by ECL.

### Animal model and treatment procedures

MHCC97H–CCN2-sh2 cells and the associated MHCC97H–Mock control cells, or MHCCC97H–LRP6-sh2 and the associated MHCC97H–Mock control cells were injected subcutaneously into the upper left flank region of 4 mice per group to produce tumors. Four weeks later, subcutaneous xenografts were measured as previously described.

In addition, 24 mice were injected with MHCC97H cells subcutaneously into the upper left flank region. Seven days later, these 24 mice were randomly divided into four groups: control group, heparin group, oxaliplatin group, and heparin + oxaliplatin group. The control group was injected with 0.1 ml 5% glucose solution (GS) and 0.1 ml 0.9% normal saline (NS) via intraperitoneal and subcutaneous injections, respectively. The heparin group was treated with 0.1 ml heparin (250 U/kg) via subcutaneous injections once a day. The oxaliplatin group was treated with 0.1 ml oxaliplatin (10 mg/kg) via intraperitoneal injection, and the heparin + oxaliplatin group was treated with both heparin and oxaliplatin as described above. Four weeks later, all subcutaneous xenografts in the four groups were measured and performed as described in previous publication [18, 37].

Finally, six mice were injected with MHCC97H–CCN2-SH2 cells subcutaneously into the upper left flank region to produce tumors. Seven days later, the six mice were randomly divided into two groups. The control group was treated with 0.1 ml 0.9% normal saline (NS), and the heparin group was treated with 0.1 ml heparin (250 U/kg). After four weeks, all subcutaneous xenografts in the two groups were measured. For additional methods please find in the Additional file 3.

## Additional files

Additional file 1: Figure S1. Expression of CCN2 and LRP6 was analyzed in 144-paired HCC samples and adjacent nontumor liver samples in training cohorts. (A) Upregulation of CCN2 in HCC samples. (B) Upregulation of LRP6 in HCC samples. **Figure S2.** Up-regulation of CCN2 and LRP6 correlates with poor prognosis and in HCC patients. Kaplan-Meier’s curves for OS and TTR according to CCN2 and LRP6 expression in the validation cohort (n=144). **Figure S3.** Expression of CCN2 and LRP6 was analyzed in 374-paired HCC samples and adjacent nontumor liver samples in validation cohorts by tissue microarrays. **Figure S4.** Oxaliplatin-treated HCC cell lines and subcutaneous tumor tissues showed increased expression of CCN2 and LRP6. (A) Upregulation of CCN2 and LRP6 in Oxaliplatin-treated HCC cell lines. (B) Upregulation of CCN2 and LRP6 in Oxaliplatin-treated subcutaneous tumor tissues. **Figure S5.** Expression of CCN2 and LRP6 from the gene expression profiles of 30-paired HCC samples with or without metastasis was analyzed. LRP6 was significantly upregulated in HCC with metastasis, while no significant association was found in the expression of CCN2. **Figure S6.** LMWH demonstrate no significant inhibitory effect on the in vitro proliferation of MHCC-97H for 24, 48, 72h, with the IC50 645±99.33, 699±87.88, and 469±72.77 U/ml respectively. **Figure S7.** The synergetic effect of LMWH combined with chemotherapy was evaluated, and LMWH (2 U/ml) significantly increased the sensitivity MHCC-97H cells to oxaliplatin. (ZIP 8918 kb)

Additional file 2: Table S1. Correlations between CCN2/LRP6 and clinicopathology feature in 374 patients with HCC. **Table S2.** Univariate analysis of factors associated with survival and recurrence in 374 patients with HCC. **Table S3.** Multivariate analysis of factors associated with survival and recurrence in 374 patients with HCC. **Table S4.** Primary antibodies used for western blot and immunohistochemistry. **Table S5.** Sequences of primers used for qRT-PCR. **Table S6.** Primers for vectors construction. **Table S7.** vshRNA target sequences for CCN2 and LRP6. (DOC 87 kb)

Additional file 3: Supplementary Materials and Methods. (DOC 47 kb)

## References

[1] Torre LA, Bray F, Siegel RL, Ferlay J, Lortet-Tieulent J, Jemal A (2015). Global cancer statistics, 2012. CA Cancer J Clin.

[2] Page AJ, Cosgrove DC, Philosophe B, Pawlik TM (2014). Hepatocellular carcinoma: diagnosis, management, and prognosis. Surg Oncol Clin N Am.

[3] Lee TK, Castilho A, Cheung VC, Tang KH, Ma S, Ng IO (2011). CD24(+) liver tumor-initiating cells drive self-renewal and tumor initiation through STAT3-mediated NANOG regulation. Cell Stem Cell.

[4] Liu LL, Fu D, Ma Y, Shen XZ (2011). The power and the promise of liver cancer stem cell markers. Stem Cells Dev.

[5] Yamashita T, Ji J, Budhu A, Forgues M, Yang W, Wang HY (2009). EpCAM-positive hepatocellular carcinoma cells are tumor-initiating cells with stem/progenitor cell features. Gastroenterology.

[6] Yang ZF, Ho DW, Ng MN, Lau CK, Yu WC, Ngai P (2008). Significance of CD90+ cancer stem cells in human liver cancer. Cancer Cell.

[7] Marquardt JU, Gomez-Quiroz L, Arreguin Camacho LO, Pinna F, Lee YH, Kitade M (2015). Curcumin effectively inhibits oncogenic NF-kappaB signaling and restrains stemness features in liver cancer. J Hepatol.

[8] Gaston-Massuet C, Andoniadou CL, Signore M, Jayakody SA, Charolidi N, Kyeyune R (2011). Increased Wingless (Wnt) signaling in pituitary progenitor/stem cells gives rise to pituitary tumors in mice and humans. Proc Natl Acad Sci U S A.

[9] Tung EK, Wong BY, Yau TO, Ng IO (2012). Upregulation of the Wnt co-receptor LRP6 promotes hepatocarcinogenesis and enhances cell invasion. PLoS One.

[10] Jia Q, Dong Q, Qin L (2016). CCN: core regulatory proteins in the microenvironment that affect the metastasis of hepatocellular carcinoma?. Oncotarget.

[11] Bradham DM, Igarashi A, Potter RL, Grotendorst GR (1991). Connective tissue growth factor: a cysteine-rich mitogen secreted by human vascular endothelial cells is related to the SRC-induced immediate early gene product CEF-10. J Cell Biol.

[12] Kothapalli D, Frazier KS, Welply A, Segarini PR, Grotendorst GR (1997). Transforming growth factor beta induces anchorage-independent growth of NRK fibroblasts via a connective tissue growth factor-dependent signaling pathway. Cell growth & differentiation : the molecular biology journal of the American Association for Cancer Research.

[13] Mason ED, Konrad KD, Webb CD, Marsh JL (1994). Dorsal midline fate in Drosophila embryos requires twisted gastrulation, a gene encoding a secreted protein related to human connective tissue growth factor. Genes Dev.

[14] Shimo T, Nakanishi T, Kimura Y, Nishida T, Ishizeki K, Matsumura T (1998). Inhibition of endogenous expression of connective tissue growth factor by its antisense oligonucleotide and antisense RNA suppresses proliferation and migration of vascular endothelial cells. J Biochem.

[15] Bu Y, Jia QA, Ren ZG, Zhang JB, Jiang XM, Liang L (2014). Maintenance of stemness in oxaliplatin-resistant hepatocellular carcinoma is associated with increased autocrine of IGF1. PLoS One.

[16] Gao R, Brigstock DR (2003). Low density lipoprotein receptor-related protein (LRP) is a heparin-dependent adhesion receptor for connective tissue growth factor (CTGF) in rat activated hepatic stellate cells. Hepatology research : the official journal of the Japan Society of Hepatology.

[17] Segarini PR, Nesbitt JE, Li D, Hays LG, Yates JR, Carmichael DF (2001). The low density lipoprotein receptor-related protein/alpha2-macroglobulin receptor is a receptor for connective tissue growth factor. J Biol Chem.

[18] Ye QH, Qin LX, Forgues M, He P, Kim JW, Peng AC (2003). Predicting hepatitis B virus-positive metastatic hepatocellular carcinomas using gene expression profiling and supervised machine learning. Nat Med.

[19] Maass T, Marquardt J, Lee JS, Krupp M, Scholz-Kreisel P, Mogler C (2016). Increased liver carcinogenesis and enrichment of stem cell properties in livers of Dickkopf 2 (Dkk2) deleted mice. Oncotarget.

[20] Wang Y, He L, Du Y, Zhu P, Huang G, Luo J (2015). The long noncoding RNA lncTCF7 promotes self-renewal of human liver cancer stem cells through activation of Wnt signaling. Cell Stem Cell.

[21] Zuo GW, Kohls CD, He BC, Chen L, Zhang W, Shi Q (2010). The CCN proteins: important signaling mediators in stem cell differentiation and tumorigenesis. Histol Histopathol.

[22] Abreu JG, Ketpura NI, Reversade B, De Robertis EM (2002). Connective-tissue growth factor (CTGF) modulates cell signalling by BMP and TGF-beta. Nat Cell Biol.

[23] Inoki I, Shiomi T, Hashimoto G, Enomoto H, Nakamura H, Makino K (2002). Connective tissue growth factor binds vascular endothelial growth factor (VEGF) and inhibits VEGF-induced angiogenesis. FASEB journal : official publication of the Federation of American Societies for Experimental Biology.

[24] Wells JE, Howlett M, Cole CH, Kees UR (2015). Deregulated expression of connective tissue growth factor (CTGF/CCN2) is linked to poor outcome in human cancer. Int J Cancer.

[25] Rooney B, O'Donovan H, Gaffney A, Browne M, Faherty N, Curran SP (2011). CTGF/CCN2 activates canonical Wnt signalling in mesangial cells through LRP6: implications for the pathogenesis of diabetic nephropathy. FEBS Lett.

[26] Perbal B (2004). CCN proteins: multifunctional signalling regulators. Lancet (London, England).

[27] Kuderer NM, Khorana AA, Lyman GH, Francis CW (2007). A meta-analysis and systematic review of the efficacy and safety of anticoagulants as cancer treatment: impact on survival and bleeding complications. Cancer.

[28] Larsen TB, Nielsen PB, Skjoth F, Rasmussen LH, Lip GY (2014). Non-vitamin K antagonist oral anticoagulants and the treatment of venous thromboembolism in cancer patients: a semi systematic review and meta-analysis of safety and efficacy outcomes. PLoS One.

[29] van Doormaal FF, Di Nisio M, Otten HM, Richel DJ, Prins M, Buller HR (2011). Randomized trial of the effect of the low molecular weight heparin nadroparin on survival in patients with cancer. Journal of clinical oncology : official journal of the American Society of Clinical Oncology.

[30] Altinbas M, Dikilitas M, Ozkan M, Dogu GG, Er O, Coskun HS (2014). The effect of small-molecular-weight heparin added to chemotherapy on survival in small-cell lung cancer - A retrospective analysis. Indian J Cancer.

[31] Park JC, Pratz CF, Tesfaye A, Brodsky RA, Antonarakis ES (2015). The effect of therapeutic anticoagulation on overall survival in men receiving first-line docetaxel chemotherapy for metastatic castration-resistant prostate cancer. Clinical genitourinary cancer.

[32] Yan J, Zheng Q, Wang Y, Lu HF, Xue Q, Tang ZY (2005). Vasoinhibitory effect of daltepartin sodium on human hepatocellular carcinoma in nude mice. Zhonghua gan zang bing za zhi = Zhonghua ganzangbing zazhi = Chinese journal of hepatology.

[33] Stolting DP, Jaehde U, Wiese M, Bendas G (2013). Are low molecular weight heparins able to sensitize chemoresistant tumor cells?. Int J Clin Pharmacol Ther.

[34] Arvatz G, Weissmann M, Ilan N, Vlodavsky I (2016). Heparanase and cancer progression: New directions, new promises. Human vaccines & immunotherapeutics.

[35] Li Y, Tang ZY, Ye SL, Liu YK, Chen J, Xue Q (2001). Establishment of cell clones with different metastatic potential from the metastatic hepatocellular carcinoma cell line MHCC97. World J Gastroenterol.

[36] Zhou H, Huang H, Shi J, Zhao Y, Dong Q, Jia H (2010). Prognostic value of interleukin 2 and interleukin 15 in peritumoral hepatic tissues for patients with hepatitis B-related hepatocellular carcinoma after curative resection. Gut.

[37] Zhang T, Sun HC, Xu Y, Zhang KZ, Wang L, Qin LX (2005). Overexpression of platelet-derived growth factor receptor alpha in endothelial cells of hepatocellular carcinoma associated with high metastatic potential. Clinical cancer research : an official journal of the American Association for Cancer Research.

