# Supplementary figures and images for: Maintenance of stemness is associated with the interation of LRP6 and heparin-binding protein CCN2 autocrined by hepatocellular carcinoma
Source: J Exp Clin Cancer Res. 2017 Sep 4;36:117. doi: 10.1186/s13046-017-0576-3 (PMC5584530; doi:10.1186/s13046-017-0576-3)

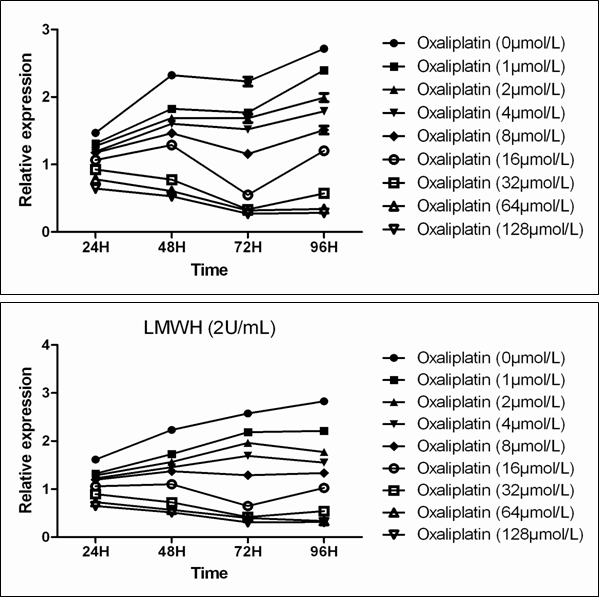

Supplement: Supplementary file 1 — Expression of CCN2 and LRP6 was analyzed in 144-paired HCC samples and adjacent nontumor liver samples in training cohorts. (A) Upregulation of CCN2 in HCC samples. (B) Upregulation of LRP6 in HCC samples. Figure S2. Up-regulation of CCN2 and LRP6 correlates with poor prognosis and in HCC patients. Kaplan-Meier’s curves for OS and TTR according to CCN2 and LRP6 expression in the validation cohort (n=144). Figure S3. Expression of CCN2 and LRP6 was analyzed in 374-paired HCC samples and adjacent nontumor liver samples in validation cohorts by tissue microarrays. Figure S4. Oxaliplatin-treated HCC cell lines and subcutaneous tumor tissues showed increased expression of CCN2 and LRP6. (A) Upregulation of CCN2 and LRP6 in Oxaliplatin-treated HCC cell lines. (B) Upregulation of CCN2 and LRP6 in Oxaliplatin-treated subcutaneous tumor tissues. Figure S5. Expression of CCN2 and LRP6 from the gene expression profiles of 30-paired HCC samples with or without metastasis was analyzed. LRP6 was significantly upregulated in HCC with metastasis, while no significant association was found in the expression of CCN2. Figure S6. LMWH demonstrate no significant inhibitory effect on the in vitro proliferation of MHCC-97H for 24, 48, 72h, with the IC50 645±99.33, 699±87.88, and 469±72.77 U/ml respectively. Figure S7. The synergetic effect of LMWH combined with chemotherapy was evaluated, and LMWH (2 U/ml) significantly increased the sensitivity MHCC-97H cells to oxaliplatin. (ZIP 8918 kb) [file 13046_2017_576_MOESM1_ESM.zip › suppl-fig7.tif]

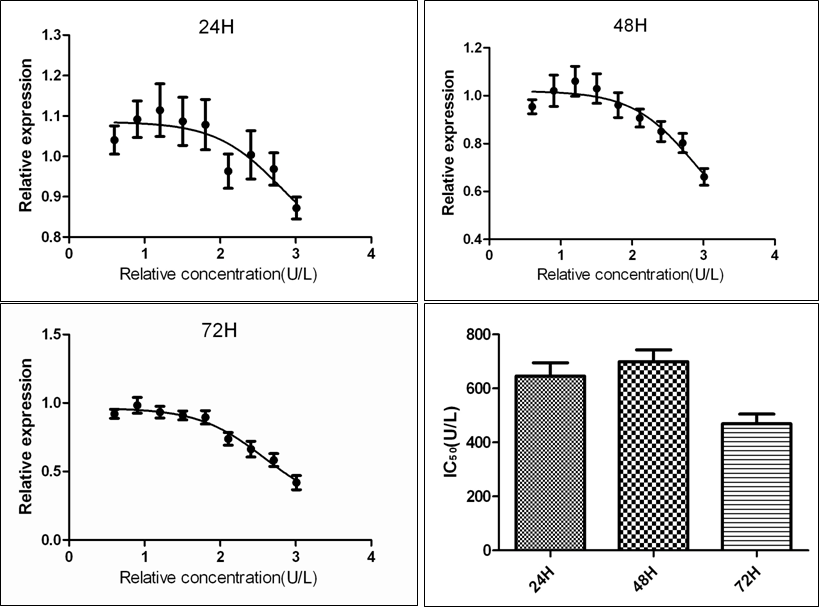

Supplement: Supplementary file 1 — Expression of CCN2 and LRP6 was analyzed in 144-paired HCC samples and adjacent nontumor liver samples in training cohorts. (A) Upregulation of CCN2 in HCC samples. (B) Upregulation of LRP6 in HCC samples. Figure S2. Up-regulation of CCN2 and LRP6 correlates with poor prognosis and in HCC patients. Kaplan-Meier’s curves for OS and TTR according to CCN2 and LRP6 expression in the validation cohort (n=144). Figure S3. Expression of CCN2 and LRP6 was analyzed in 374-paired HCC samples and adjacent nontumor liver samples in validation cohorts by tissue microarrays. Figure S4. Oxaliplatin-treated HCC cell lines and subcutaneous tumor tissues showed increased expression of CCN2 and LRP6. (A) Upregulation of CCN2 and LRP6 in Oxaliplatin-treated HCC cell lines. (B) Upregulation of CCN2 and LRP6 in Oxaliplatin-treated subcutaneous tumor tissues. Figure S5. Expression of CCN2 and LRP6 from the gene expression profiles of 30-paired HCC samples with or without metastasis was analyzed. LRP6 was significantly upregulated in HCC with metastasis, while no significant association was found in the expression of CCN2. Figure S6. LMWH demonstrate no significant inhibitory effect on the in vitro proliferation of MHCC-97H for 24, 48, 72h, with the IC50 645±99.33, 699±87.88, and 469±72.77 U/ml respectively. Figure S7. The synergetic effect of LMWH combined with chemotherapy was evaluated, and LMWH (2 U/ml) significantly increased the sensitivity MHCC-97H cells to oxaliplatin. (ZIP 8918 kb) [file 13046_2017_576_MOESM1_ESM.zip › suppl-fig6.tif]

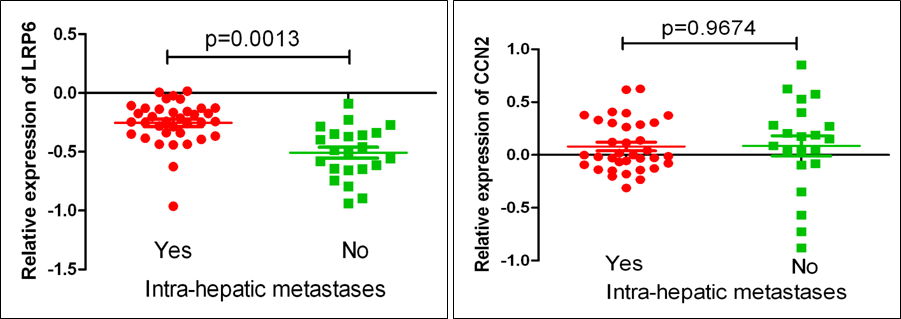

Supplement: Supplementary file 1 — Expression of CCN2 and LRP6 was analyzed in 144-paired HCC samples and adjacent nontumor liver samples in training cohorts. (A) Upregulation of CCN2 in HCC samples. (B) Upregulation of LRP6 in HCC samples. Figure S2. Up-regulation of CCN2 and LRP6 correlates with poor prognosis and in HCC patients. Kaplan-Meier’s curves for OS and TTR according to CCN2 and LRP6 expression in the validation cohort (n=144). Figure S3. Expression of CCN2 and LRP6 was analyzed in 374-paired HCC samples and adjacent nontumor liver samples in validation cohorts by tissue microarrays. Figure S4. Oxaliplatin-treated HCC cell lines and subcutaneous tumor tissues showed increased expression of CCN2 and LRP6. (A) Upregulation of CCN2 and LRP6 in Oxaliplatin-treated HCC cell lines. (B) Upregulation of CCN2 and LRP6 in Oxaliplatin-treated subcutaneous tumor tissues. Figure S5. Expression of CCN2 and LRP6 from the gene expression profiles of 30-paired HCC samples with or without metastasis was analyzed. LRP6 was significantly upregulated in HCC with metastasis, while no significant association was found in the expression of CCN2. Figure S6. LMWH demonstrate no significant inhibitory effect on the in vitro proliferation of MHCC-97H for 24, 48, 72h, with the IC50 645±99.33, 699±87.88, and 469±72.77 U/ml respectively. Figure S7. The synergetic effect of LMWH combined with chemotherapy was evaluated, and LMWH (2 U/ml) significantly increased the sensitivity MHCC-97H cells to oxaliplatin. (ZIP 8918 kb) [file 13046_2017_576_MOESM1_ESM.zip › suppl-fig5.tif]

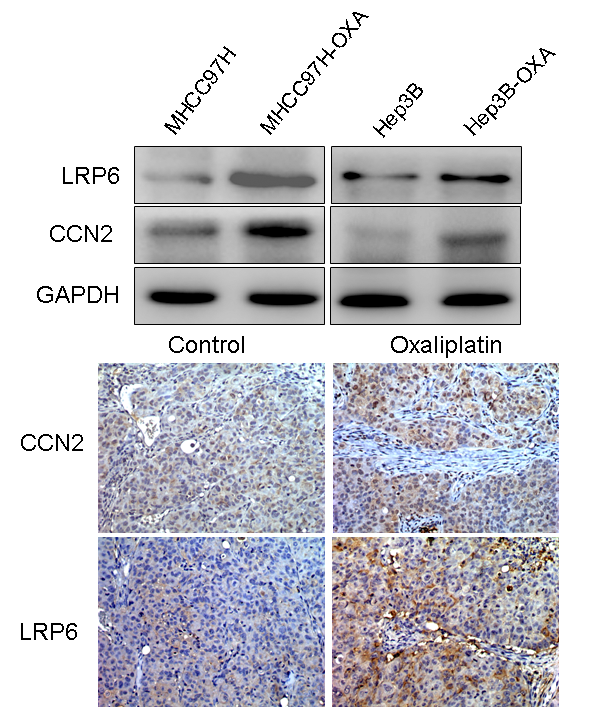

Supplement: Supplementary file 1 — Expression of CCN2 and LRP6 was analyzed in 144-paired HCC samples and adjacent nontumor liver samples in training cohorts. (A) Upregulation of CCN2 in HCC samples. (B) Upregulation of LRP6 in HCC samples. Figure S2. Up-regulation of CCN2 and LRP6 correlates with poor prognosis and in HCC patients. Kaplan-Meier’s curves for OS and TTR according to CCN2 and LRP6 expression in the validation cohort (n=144). Figure S3. Expression of CCN2 and LRP6 was analyzed in 374-paired HCC samples and adjacent nontumor liver samples in validation cohorts by tissue microarrays. Figure S4. Oxaliplatin-treated HCC cell lines and subcutaneous tumor tissues showed increased expression of CCN2 and LRP6. (A) Upregulation of CCN2 and LRP6 in Oxaliplatin-treated HCC cell lines. (B) Upregulation of CCN2 and LRP6 in Oxaliplatin-treated subcutaneous tumor tissues. Figure S5. Expression of CCN2 and LRP6 from the gene expression profiles of 30-paired HCC samples with or without metastasis was analyzed. LRP6 was significantly upregulated in HCC with metastasis, while no significant association was found in the expression of CCN2. Figure S6. LMWH demonstrate no significant inhibitory effect on the in vitro proliferation of MHCC-97H for 24, 48, 72h, with the IC50 645±99.33, 699±87.88, and 469±72.77 U/ml respectively. Figure S7. The synergetic effect of LMWH combined with chemotherapy was evaluated, and LMWH (2 U/ml) significantly increased the sensitivity MHCC-97H cells to oxaliplatin. (ZIP 8918 kb) [file 13046_2017_576_MOESM1_ESM.zip › suppl-fig4.tif]

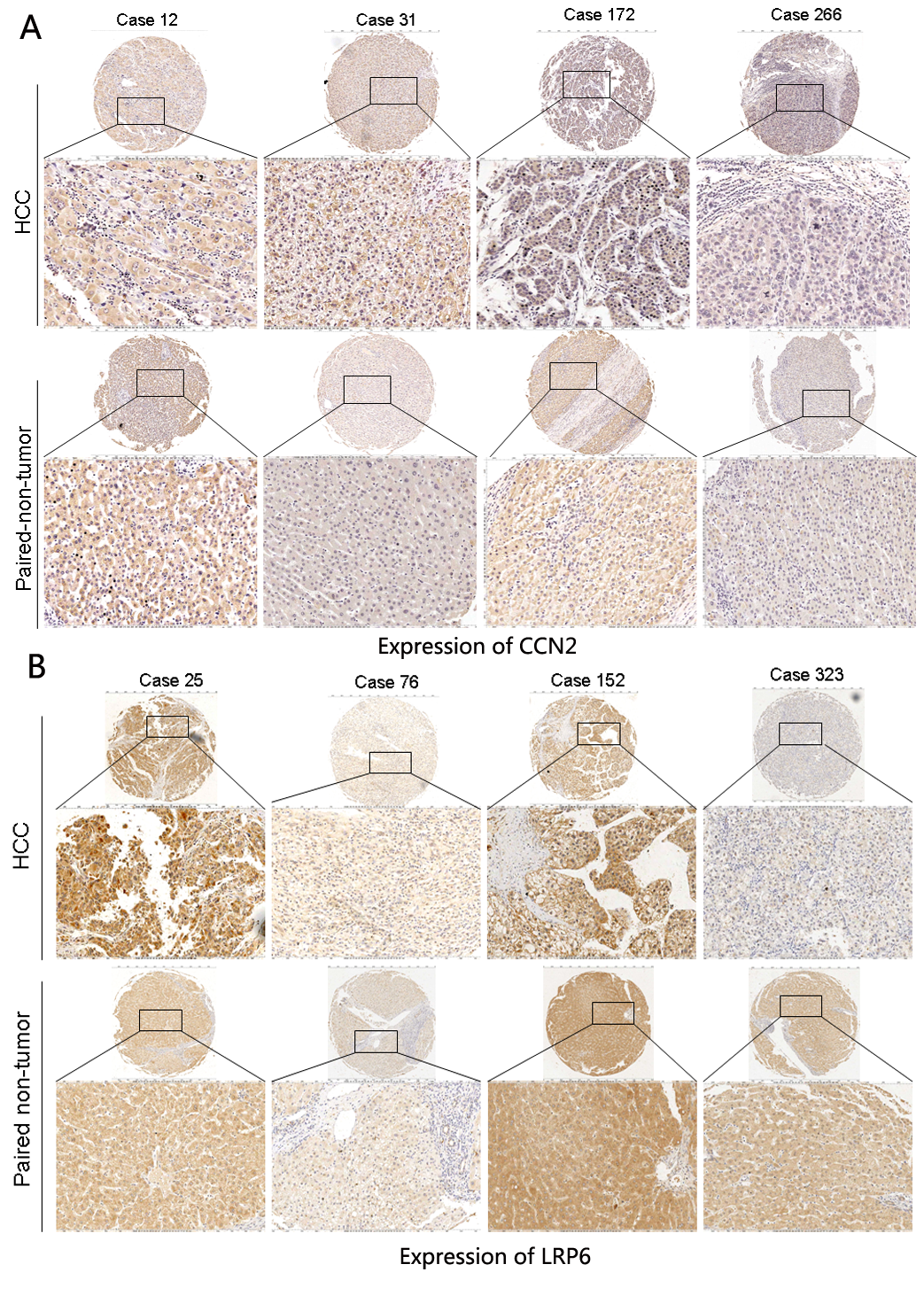

Supplement: Supplementary file 1 — Expression of CCN2 and LRP6 was analyzed in 144-paired HCC samples and adjacent nontumor liver samples in training cohorts. (A) Upregulation of CCN2 in HCC samples. (B) Upregulation of LRP6 in HCC samples. Figure S2. Up-regulation of CCN2 and LRP6 correlates with poor prognosis and in HCC patients. Kaplan-Meier’s curves for OS and TTR according to CCN2 and LRP6 expression in the validation cohort (n=144). Figure S3. Expression of CCN2 and LRP6 was analyzed in 374-paired HCC samples and adjacent nontumor liver samples in validation cohorts by tissue microarrays. Figure S4. Oxaliplatin-treated HCC cell lines and subcutaneous tumor tissues showed increased expression of CCN2 and LRP6. (A) Upregulation of CCN2 and LRP6 in Oxaliplatin-treated HCC cell lines. (B) Upregulation of CCN2 and LRP6 in Oxaliplatin-treated subcutaneous tumor tissues. Figure S5. Expression of CCN2 and LRP6 from the gene expression profiles of 30-paired HCC samples with or without metastasis was analyzed. LRP6 was significantly upregulated in HCC with metastasis, while no significant association was found in the expression of CCN2. Figure S6. LMWH demonstrate no significant inhibitory effect on the in vitro proliferation of MHCC-97H for 24, 48, 72h, with the IC50 645±99.33, 699±87.88, and 469±72.77 U/ml respectively. Figure S7. The synergetic effect of LMWH combined with chemotherapy was evaluated, and LMWH (2 U/ml) significantly increased the sensitivity MHCC-97H cells to oxaliplatin. (ZIP 8918 kb) [file 13046_2017_576_MOESM1_ESM.zip › suppl-fig3.tif]

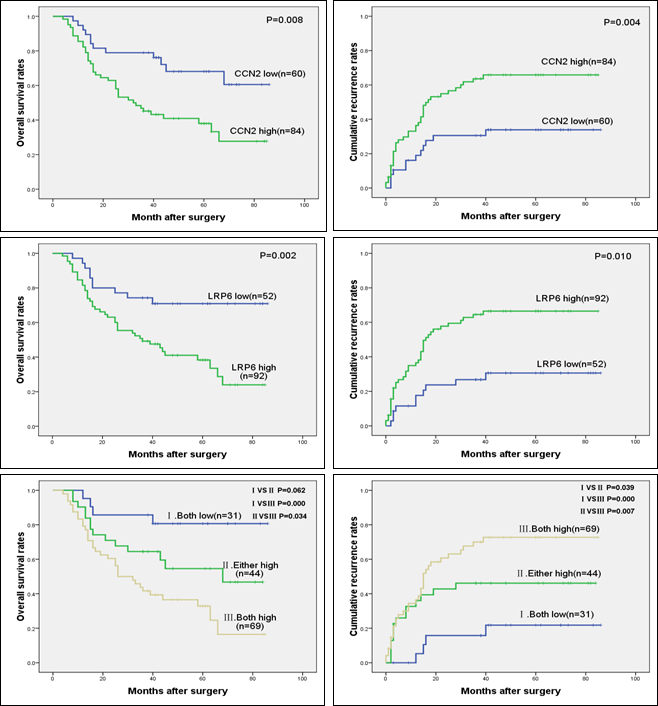

Supplement: Supplementary file 1 — Expression of CCN2 and LRP6 was analyzed in 144-paired HCC samples and adjacent nontumor liver samples in training cohorts. (A) Upregulation of CCN2 in HCC samples. (B) Upregulation of LRP6 in HCC samples. Figure S2. Up-regulation of CCN2 and LRP6 correlates with poor prognosis and in HCC patients. Kaplan-Meier’s curves for OS and TTR according to CCN2 and LRP6 expression in the validation cohort (n=144). Figure S3. Expression of CCN2 and LRP6 was analyzed in 374-paired HCC samples and adjacent nontumor liver samples in validation cohorts by tissue microarrays. Figure S4. Oxaliplatin-treated HCC cell lines and subcutaneous tumor tissues showed increased expression of CCN2 and LRP6. (A) Upregulation of CCN2 and LRP6 in Oxaliplatin-treated HCC cell lines. (B) Upregulation of CCN2 and LRP6 in Oxaliplatin-treated subcutaneous tumor tissues. Figure S5. Expression of CCN2 and LRP6 from the gene expression profiles of 30-paired HCC samples with or without metastasis was analyzed. LRP6 was significantly upregulated in HCC with metastasis, while no significant association was found in the expression of CCN2. Figure S6. LMWH demonstrate no significant inhibitory effect on the in vitro proliferation of MHCC-97H for 24, 48, 72h, with the IC50 645±99.33, 699±87.88, and 469±72.77 U/ml respectively. Figure S7. The synergetic effect of LMWH combined with chemotherapy was evaluated, and LMWH (2 U/ml) significantly increased the sensitivity MHCC-97H cells to oxaliplatin. (ZIP 8918 kb) [file 13046_2017_576_MOESM1_ESM.zip › suppl-fig2.tif]

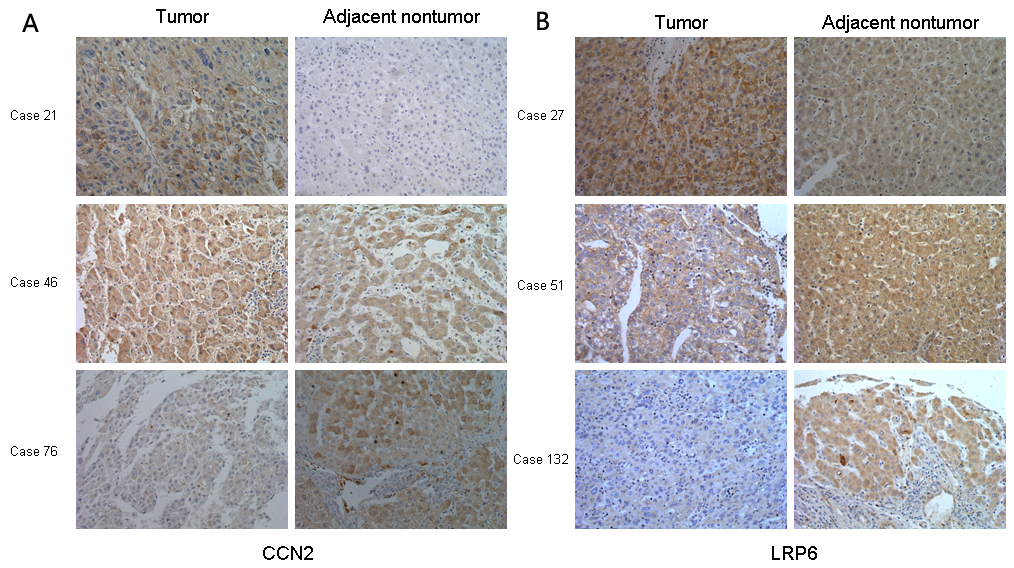

Supplement: Supplementary file 1 — Expression of CCN2 and LRP6 was analyzed in 144-paired HCC samples and adjacent nontumor liver samples in training cohorts. (A) Upregulation of CCN2 in HCC samples. (B) Upregulation of LRP6 in HCC samples. Figure S2. Up-regulation of CCN2 and LRP6 correlates with poor prognosis and in HCC patients. Kaplan-Meier’s curves for OS and TTR according to CCN2 and LRP6 expression in the validation cohort (n=144). Figure S3. Expression of CCN2 and LRP6 was analyzed in 374-paired HCC samples and adjacent nontumor liver samples in validation cohorts by tissue microarrays. Figure S4. Oxaliplatin-treated HCC cell lines and subcutaneous tumor tissues showed increased expression of CCN2 and LRP6. (A) Upregulation of CCN2 and LRP6 in Oxaliplatin-treated HCC cell lines. (B) Upregulation of CCN2 and LRP6 in Oxaliplatin-treated subcutaneous tumor tissues. Figure S5. Expression of CCN2 and LRP6 from the gene expression profiles of 30-paired HCC samples with or without metastasis was analyzed. LRP6 was significantly upregulated in HCC with metastasis, while no significant association was found in the expression of CCN2. Figure S6. LMWH demonstrate no significant inhibitory effect on the in vitro proliferation of MHCC-97H for 24, 48, 72h, with the IC50 645±99.33, 699±87.88, and 469±72.77 U/ml respectively. Figure S7. The synergetic effect of LMWH combined with chemotherapy was evaluated, and LMWH (2 U/ml) significantly increased the sensitivity MHCC-97H cells to oxaliplatin. (ZIP 8918 kb) [file 13046_2017_576_MOESM1_ESM.zip › suppl-fig1.tif]
