# Supplementary material for: Maintenance of stemness is associated with the interation of LRP6 and heparin-binding protein CCN2 autocrined by hepatocellular carcinoma
Source: J Exp Clin Cancer Res. 2017 Sep 4;36:117. doi: 10.1186/s13046-017-0576-3 (PMC5584530; doi:10.1186/s13046-017-0576-3)
Supplement: Supplementary file 2 — Correlations between CCN2/LRP6 and clinicopathology feature in 374 patients with HCC. Table S2. Univariate analysis of factors associated with survival and recurrence in 374 patients with HCC. Table S3. Multivariate analysis of factors associated with survival and recurrence in 374 patients with HCC. Table S4. Primary antibodies used for western blot and immunohistochemistry. Table S5. Sequences of primers used for qRT-PCR. Table S6. Primers for vectors construction. Table S7. vshRNA target sequences for CCN2 and LRP6. (DOC 87 kb) [file 13046_2017_576_MOESM2_ESM.doc]

Table S1 Correlations between CCN2/LRP6 and clinicopathology feature in 374 patients with HCC

| Variable | No. of Patient | |  | No. of Patient | |  |
| --- | --- | --- | --- | --- | --- | --- |
|  | CCN2^high^ | CCN2^low^ | *p* | LRP6 ^high^ | LRP6 ^low^ | *p* |
| Age, y  ≥53  ＜53 | 71  108 | 92  103 | 0.087 | 98  124 | 65  87 | 0.437 |
| Sex  Men  Women | 150  29 | 156  39 | 0.207 | 184  38 | 122  30 | 0.304 |
| HBsAg  Positive  Negative | 167  12 | 180  15 | 0.434 | 208  14 | 139  13 | 0.265 |
| Cirrhosis  Yes  no | 158  21 | 162  33 | 0.100 | 194  28 | 126  26 | 0.144 |
| Serum, AFP  ≥20  ＜20 | 116  63 | 121  74 | 0.328 | 142  80 | 95  57 | 0.428 |
| Serum, ALT  ≥75  ＜75 | 15  164 | 24  171 | 0.142 | 24  198 | 15  137 | 0.455 |
| Tumor dimension  ≥5cm  ＜5cm | 42  137 | 38  157 | 0.209 | 51  171 | 29  123 | 0.220 |
| No. of tumors  Multiple  single | 13  166 | 5  190 | **0.029** | 14  208 | 4  148 | 0.140* |
| Vascular invasion  Yes  No | 62  117 | 48  147 | **0.022** | 75  147 | 35  117 | **0.016** |
| Tumor encapsulation  Complete  None | 93  86 | 80  115 | **0.034** | 94  128 | 79  73 | **0.042** |

AFP, alpha-fetoprotein; HBsAg, hepatitis B surface antigen

*Fisher exact test

Table S2 Univariate Analysis of Factors Associated With Survival and Recurrence (n=374)

| Variables | Overall Survival | | Cumulative Recurrence | |
| --- | --- | --- | --- | --- |
|  | Hazard ratio (95%  confidence interval)*a* | *P* value | Hazard ratio (95%  confidence interval)*a* | *P* value |
| Age, *y*, ≥53 vs ＜53 | .573 (.383- 1.459) | 0.412 | .516(.347- 1.274) | 0.515 |
| Sex, male vs female | .865 (.420-2.524) | 1.071 | 1.144 (.444-2.519) | 0.921 |
| HBsAg, positive VS negative | 1.617 (.601-2.856) | 0.619 | 1.315(.602-2.595) | 0.549 |
| Cirrhosis, no vs yes | 1.580 (1.173-2.342) | 0.089 | .729（.354-1.413） | 0.233 |
| AFP, *ng/mL*, ≥20 vs ＜20 | 2.540 (1.266-5.098) | 0.539 | 2.580（1.380-5.313） | **0.033** |
| Tumor size | 1.378（1.118-1.663） | **0.026** | 1.683（1.003- 2.134） | **0.019** |
| No. of tumors | 2.178 (1.201-3.952) | **0.010** | 2.064（1.251-4.137） | **0.007** |
| Vascular invasion, yes vs no | 2.477(1.491-5.537) | **0.003** | 2.191（1.422-3.661） | **0.002** |
| LRP6, high vs low | 3.019（1.298- 5.981） | **0.000** | 2.383（1.444- 5.788） | **0.000** |
| CCN2, high vs low | 2.853 (1.398-5.310) | **0.001** | 2.392（1.245- 4.596） | **0.004** |

Table S3 Multivarite Analysis of Factors Associated With Survival and Recurrence (n=374)

| Variables | Overall Survival | | Cumulative Recurrence | |
| --- | --- | --- | --- | --- |
|  | Hazard ratio (95%  confidence interval)*a* | *P* value | Hazard ratio (95%  confidence interval)*a* | *P* value |
| AFP, ng/mL, ≥20 vs ＜20 | 2.460 (1.051- 4.352) | 0.282 | 2.493（1.210- 5.345） | **0.039** |
| Tumor size | 1.512 (1.233- 1.978) | **0.013** | 1.124（1.023- 1.199） | **0.006** |
| No. of tumors | 1.481 (.812- 2.973) | 0.183 | 1.592（.890- 3.116） | 0.106 |
| Vascular invasion, yes vs no | 2.082 (1.144- 3.931) | **0.019** | 1.873（.885- 3.312） | **0.041** |
| CCN2, high vs low | 1.899 (1.128- 4.289) | **0.002** | 1.685（.713- 3.516） | **0.018** |
| LRP6, high vs low | 3.665（1.355-7.881） | **0.006** | 2.392（1.007- 5.486） | **0.014** |

Table S4 Primary Antibodies for Western Blot, Immunohistochemistry

| Antibody | Concentration for WB | Concentration for IHC | Specifity | Company |
| --- | --- | --- | --- | --- |
| LRP6 | 1:1000 | 1:100 | Rabbit monoclonal | Abcam |
| p-LRP6 | 1:800 | - | Rabbit monoclonal | Abcam |
| CCN2 | 1:1000 | 1:500 | Rabbit polyclonal | Abcam |
| P-β catenin (Tyr142) | 1:1000 | - | Rabbit polyclonal | CST |
| β catenin | 1:600 | - | Rabbit monoclonal | Abcam |
| P-GSK3β(Ser 9) | 1:400 | - | Rabbit monoclonal | CST |
| GSK3β  E-cadherin | 1:2000  1:1000 | -  - | Rabbit monoclonal  Rabbit monoclonal | CST  Abcam |
| CD90 | 1:1000 | - | Rabbit monoclonal | Abcam |
| SOX2 | 1:200 | - | Rabbit polyclonal | Epitomics |
| GAPDH | 1:1000 | - | Mouse monoclonal | Abcam |
| β-actin | 1:1000 | - | Mouse monoclonal | Abcam |

Table S5 Primers for qRT-PCR

| Gene | Forward primer (5’---3’) | Reverse primer (5’---3’) | Melting temperature | |
| --- | --- | --- | --- | --- |
| LRP6 | TGGGCTCAACCGTGAAGTTATA | CGAGATGAGTGAAAGACGAGGA | 60 | 195bp |
| CCN2 | GCATCTTCGGTGGTACGGTGTA | TGGACCAGGCAGTTGGCTCTA | 60 | 298bp |
| Actin | CACCCAGCACAATGAAGATCAAGAT | CCAGTTTTTAAATCCTGAGTCAAGC | 60 | 317bp |

Table S6 Primers for vectors construction

| Gene | Forward primer (5’---3’) | Reverse primer (5’---3’) |
| --- | --- | --- |
| LRP6 | TAGAGCTAGCGAATTATGGGGGCCGTCCTGA | AGATCCTTCGCGGCCTCAGGAGGAGTCTGTAC |
| CCN2 | CATAGAAGATTCTAGATGACCGCCGCCAGTATGG | ATTTAAATTCGAATTTCATGCCATGTCTCCGTACAT |

Table S7 vshRNA Target Sequences

|  | Target sequence |
| --- | --- |
| GFP-LRP6 |  |
| VshRNA1 | GATGCAATGGAGATGCAAA |
| VshRNA2 | GCGAATTGAAAGCAGTGAT |
| VshRNA3 | TGCCCACTACTCTCTTAAT |
| Puro-CCN2 |  |
| VshRNA1 | ATGTCAAACAAATAGTCTATC |
| VshRNA2 | CATCTTTGAATCGCTGTACTA |
| VshRNA3 | GCATGAAGACATACCGAGCTA |
