# Supplementary material for: Maintenance of stemness is associated with the interation of LRP6 and heparin-binding protein CCN2 autocrined by hepatocellular carcinoma
Source: J Exp Clin Cancer Res. 2017 Sep 4;36:117. doi: 10.1186/s13046-017-0576-3 (PMC5584530; doi:10.1186/s13046-017-0576-3)
Supplement: Supplementary file 3 — Supplementary Materials and Methods. (DOC 47 kb) [file 13046_2017_576_MOESM3_ESM.doc]

**Supplementary Materials and Methods**

**Reagents and Antibodies.** Sodium chlorate (NaClO_3_), LMWH, heparinase, and oxaliplatin were obtained from Sigma (St. Louis, MO, USA). Cell Counting Kit 8 was obtained from Dojindo (Kumamoto, Japan). LMWHs was obtained from Pfizer. Combinant human CTGF and DKK-1 were obtained from Peprotech (Rocky Hill, NJ, USA). The primary antibodies used in this study are listed in Supplementary Tab. 4.

**Construction of Tissue Microarrays, Immunohistochemistry, and Immunoblot.** Tissue Microarrays (TMAs) were constructed by Shanghai Biochip Co, Ltd. as previously described [1]. Paraffin-embedded tissue sections (4 μm) were prepared according to classic methods. Immunohistochemistry and Western blot was performed as previously described [2]. The concentration of extracted protein was determined using the BCA Protein Assay Kit (Beyotime. Shanghai, China). The concentrations of primary antibodies used in these studies are also listed in Supplementary Tab. 4.

**RNA Extraction and qRT-PCR.** Total RNA was extracted from the human HCC tissues, adjacent nontumor liver samples, and the HCC cell line using the Trizol reagent (Invitrogen, Carlsbad, CA, USA). The primers used for the amplification of human genes are shown in Supplementary Tab. 5.

**Cell Proliferation Assay.** The MHCC97H cells were cultured in 96-well plates (4 × 10^3^ cells/well) and exposed to increasing concentrations of oxaliplatin (0, 0.5, 2, 4, 8, 16, 32, 64, and 128 μmol/L) or identical increasing concentrations of oxaliplatin combined with heparin (2 U/ml) for 24, 48, 72, and 96 h. The relative number of cells was carried out with the Cell Counting Kit 8 (Dojindo). Results were expressed as the absorbance of each well at 450 nm (OD 450). Proliferation ability were also detected by adherent colony experiments as we previously described [3].

**Cell Migration, Matrigel Invasion, and Adherent Colony Formation and Sphere** **Formation Ability Assays.** Cell migration, invasion and colony formation assays was performed as previously described [3, 4].

**Statistical Analysis.**

Kaplan-Meier analysis was performed to compare CCR and OS between patients in different groups and statistical p values were generated by the Cox-Mantel log-rank test. Univariate and multivariate analyses were based on the Cox proportional hazards regression model. Quantitative differences in the data on tumor volume, gene and protein expression levels, cell invasiveness, colony formation, and oxaliplatin sensitivity were evaluated by t-test. Statistical analyses were performed using SPSS 15.0 for Windows (SPSS) as previously described [1]. A p-value of less than 0.05 was considered statistically significant.

**Reference**

1. Ke AW, Shi GM, Zhou J, Wu FZ, Ding ZB, Hu MY, Xu Y, Song ZJ, Wang ZJ, Wu JC et al: Role of overexpression of CD151 and/or c-Met in predicting prognosis of hepatocellular carcinoma. Hepatology 2009, 49(2):491-503.

2. Jia QA, Ren ZG, Bu Y, Wang ZM, Zhang QB, Liang L, Jiang XM, Tang ZY: Herbal Compound "Songyou Yin" Renders Hepatocellular Carcinoma Sensitive to Oxaliplatin through Inhibition of Stemness. Evid Based Complement Alternat Med 2012, 2012:908601.

3. Bu Y, Jia QA, Ren ZG, Xue TC, Zhang QB, Zhang KZ, You Y, Tian H, Qin LX, Tang ZY: The herbal compound Songyou Yin (SYY) inhibits hepatocellular carcinoma growth and improves survival in models of chronic fibrosis via paracrine inhibition of activated hepatic stellate cells. Oncotarget 2015.

4. Wan S, Zhao E, Kryczek I, Vatan L, Sadovskaya A, Ludema G, Simeone DM, Zou W, Welling TH: Tumorassociated macrophages produce interleukin 6 and signal via STAT3 to promote expansion of human hepatocellular carcinoma stem cells. Gastroenterology 2014, 147(6):1393-1404.
